# A review of bioengineering techniques applied to breast tissue: Mechanical properties, tissue engineering and finite element analysis

**DOI:** 10.3389/fbioe.2023.1161815

**Published:** 2023-04-03

**Authors:** Ana Margarida Teixeira, Pedro Martins

**Affiliations:** ^1^ UBS, INEGI, LAETA, Porto, Portugal; ^2^ I3A, Universidad de Zaragoza, Zaragoza, Spain

**Keywords:** breast tissues, mechanical properties, 3D bioprinting, scaffolds, hydrogels, finite element modeling

## Abstract

Female breast cancer was the most prevalent cancer worldwide in 2020, according to the Global Cancer Observatory. As a prophylactic measure or as a treatment, mastectomy and lumpectomy are often performed at women. Following these surgeries, women normally do a breast reconstruction to minimize the impact on their physical appearance and, hence, on their mental health, associated with self-image issues. Nowadays, breast reconstruction is based on autologous tissues or implants, which both have disadvantages, such as volume loss over time or capsular contracture, respectively. Tissue engineering and regenerative medicine can bring better solutions and overcome these current limitations. Even though more knowledge needs to be acquired, the combination of biomaterial scaffolds and autologous cells appears to be a promising approach for breast reconstruction. With the growth and improvement of additive manufacturing, three dimensional (3D) printing has been demonstrating a lot of potential to produce complex scaffolds with high resolution. Natural and synthetic materials have been studied in this context and seeded mainly with adipose derived stem cells (ADSCs) since they have a high capability of differentiation. The scaffold must mimic the environment of the extracellular matrix (ECM) of the native tissue, being a structural support for cells to adhere, proliferate and migrate. Hydrogels (e.g., gelatin, alginate, collagen, and fibrin) have been a biomaterial widely studied for this purpose since their matrix resembles the natural ECM of the native tissues. A powerful tool that can be used in parallel with experimental techniques is finite element (FE) modeling, which can aid the measurement of mechanical properties of either breast tissues or scaffolds. FE models may help in the simulation of the whole breast or scaffold under different conditions, predicting what might happen in real life. Therefore, this review gives an overall summary concerning the human breast, specifically its mechanical properties using experimental and FE analysis, and the tissue engineering approaches to regenerate this particular tissue, along with FE models.

## 1 Introduction

The breast is a vital organ, especially for women. It has a heterogeneous structure, composed of adipose, glandular and fibrous tissues, and suspensory ligaments. Its morphology and structure, and consequently the mechanical properties change along the life of women due to many factors, such as age, hormonal state, menopause, menstrual cycle, pregnancy, and lactation, or in a presence of a pathology (Ramião et al., 2016).

Female breast cancer was the most prevalent cancer in 2020 worldwide, with 2.261.419 new cases according to the Global Cancer Observatory (Sung et al., 2021). Frequently to prevent or treat breast cancer, women undergo a mastectomy or a lumpectomy (Rocco et al., 2016). It is estimated that 28%–60% of breast cancer cases require a mastectomy, where the entire breast is removed (Cleversey et al., 2019). In a lumpectomy, only the regions with a tumor and the surrounding tissue are removed (Babarenda Gamage et al., 2017). Both surgeries, but especially mastectomy, affect the appearance of the woman and, hence, her mental health (O’Halloran et al., 2017; Donnely et al., 2020). Some studies pointed to physical attractiveness as the main body-related concern, shown to be directly associated with mental health, being depression and anxiety the two most prevalent mental disorders (Heidari et al., 2015; Tsaras et al., 2018). Therefore, breast reconstruction is usually the following medical procedure in order to recover the breast shape and volume (Rocco et al., 2016; Calvo-Gallego et al., 2020) and, hence, improve the psychological state of the woman (Combellack et al., 2016; Visscher et al., 2017; Chae et al., 2018; Rocco et al., 2019).

**FIGURE 1:**
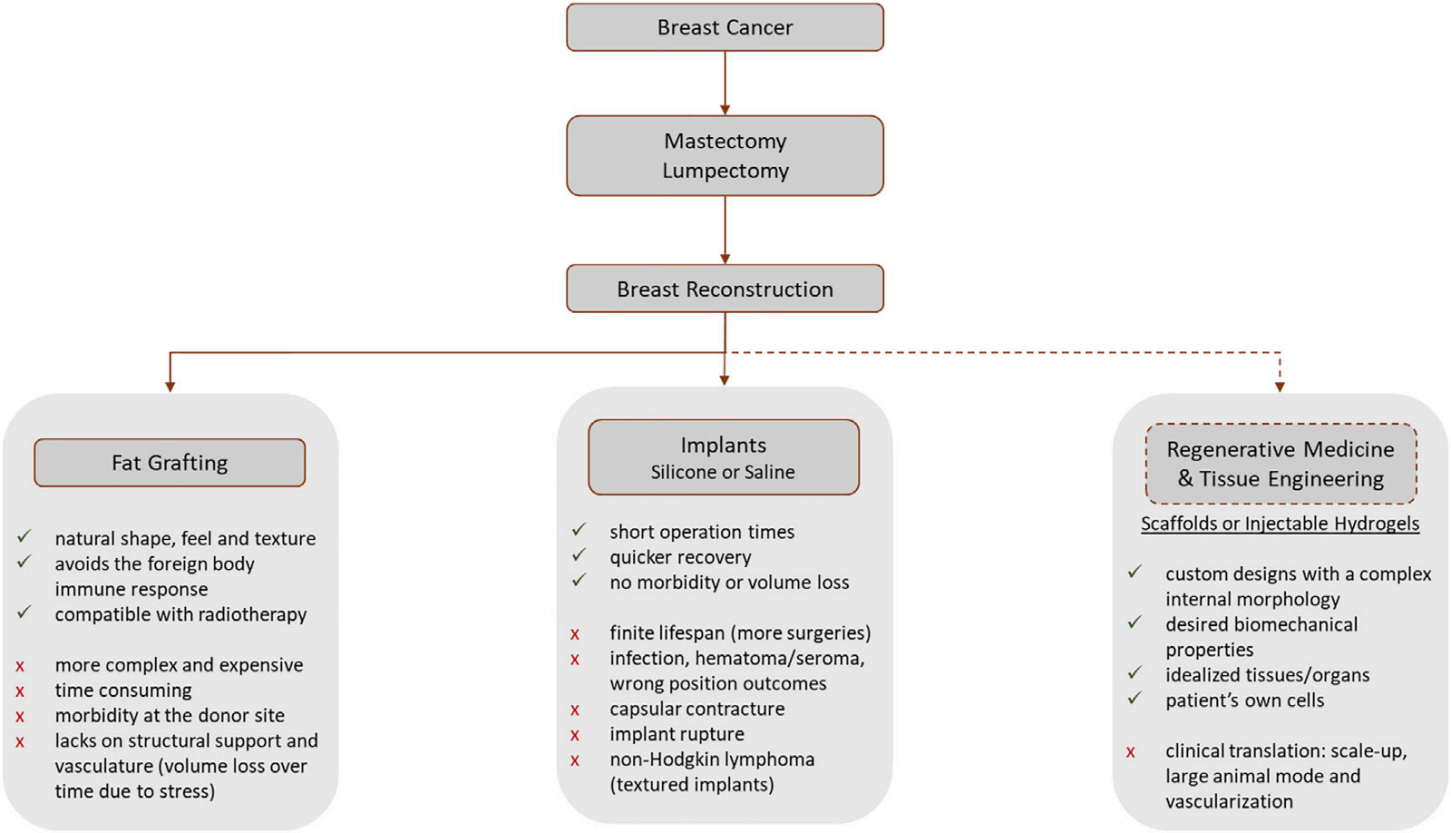
Workflow representing the most common post-breast cancer diagnosis steps that involve a mastectomy or lumpectomy. The advantages and disadvantages of each procedure are presented (Patrick, 2004; Combellack et al., 2016; Rocco et al., 2016; Visscher et al., 2017; O’Halloran N. A. et al., 2018; Chae et al., 2018; Mohseni et al., 2018; Cleversey et al., 2019; Rocco et al., 2019; Donnely et al., 2020; Janzekovic et al., 2020). The dashed line represents the research-only procedures, while the full lines represent the current clinically available treatments.

Besides the cancer treatment, breast reconstruction gained the spotlight for the efforts over the post-surgical quality of life improvement (Na et al., 2019), with a positive impact on patient’s psycho-social outcomes (O’Halloran N. A. et al., 2018; Calvo-Gallego et al., 2020). To mitigate the effects of mastectomy or for aesthetic purposes, the use of mammary prostheses is a worldwide reality, especially in western countries. Breast augmentation, including saline and silicone implants and fat transfer, was the most performed aesthetical surgical procedure worldwide in 2021, for women, 2020 with 1.658.673 surgeries 1.624.281 surgeries, corresponding to 16.0% of the total surgical procedures (ISAPS, 2021). Nowadays, breast reconstruction is performed using autologous tissues and implants (Visscher et al., 2017; O’Halloran N. A. et al., 2018; Cleversey et al., 2019) (Figure 1). In autologous reconstruction, the breast is replaced by own patient’s tissues, such as skin, fat, and muscle, from another body region (Cleversey et al., 2019). In an implant-based approach, a saline or silicone implant or biological matrices are often used.

Fat grafting is one example of an autologous technique where autologous adipose tissue (isolated from a donor site *via* liposuction) is injected into the breast. However, it lacks structural support and vasculature, which results overtime in a stress-induced volume loss of 20%–70% due to the applied forces (Visscher et al., 2017), requiring additional lipotransfer sessions to obtain the desired outcomes (Chhaya et al., 2016; O’Halloran N. A. et al., 2018). Even though autologous reconstruction results in a more natural shape, feel, and texture, avoids the foreign body immune response, and is compatible with radiotherapy (Combellack et al., 2016), this technique is more complex, time-consuming, expensive, and causes morbidity at the donor site (O’Halloran N. A. et al., 2018; Cleversey et al., 2019; Janzekovic et al., 2020), not being suitable for large defects due to the lack of adequate vasculature (Chhaya et al., 2016; Mohseni et al., 2018). However, solutions have been studied in order to enrich the fat graft with autologous ADSCs (Cleversey et al., 2019).

On the other hand, reconstruction with implants has the advantages of shorter operation times, and quicker return to normal activities, without concern over donor site morbidity and volume loss (Rocco et al., 2016, 2019; O’Halloran N. A. et al., 2018). Therefore, nowadays implant-based reconstruction is preferred over autologous-based reconstruction (O’Halloran et al., 2017). The major problem of silicone implants is the capsular contracture (e.g., capsule thickening and contraction), resulting from the failure of the normal healing process causing an excessive fibrotic reaction (Chhaya et al., 2016; Visscher et al., 2017; Rocco et al., 2019). This condition is painful and causes discomfort for women and may induce distortion of the implant and the breast. It was found that capsule contracture and capsule stiffness, are related to capsule thickening (which increases over time), alignment of the collagen fibers, and presence of contractile myofibroblasts (Bui et al., 2015; Rocco et al., 2016; O’Halloran N. et al., 2018). To overcome this drawback, modifications on the implant surfaces, such as a rough textured surface or a polyurethane coating, and the combination of implant reconstruction with autologous fat grafting could be taken into consideration (O’Halloran N. A. et al., 2018).

It was proven that silicone implants have a finite lifespan (i.e. 10 years) and possible failure (Rohrich et al., 1998), leading to additional surgeries, with added risks and costs for the patient (Visscher et al., 2017). It is estimated that within 5 years of reconstruction, these patients face a 40% re-operation rate due to short-term complications (such as infection, hematoma or seroma formation, asymmetrical or wrong position outcomes) or long-term complications (such as capsular contracture and implant rupture) (Visscher et al., 2017; O’Halloran N. A. et al., 2018; Cleversey et al., 2019; Donnely et al., 2020; Janzekovic et al., 2020). Those complications have a higher incidence if patients need to do radiotherapy after reconstruction (Visscher et al., 2017). Moreover, textured implants are being investigated due to the possible relation to anaplastic large cell lymphoma, called non-Hodgkin lymphoma, (O’Halloran N. A. et al., 2018; Donnely et al., 2020).

PIP (Poly Implant Prothèse) implants have been previously investigated by our research team to study the rupture of the silicone shells. Striations were found, indicating the occurrence of fatigue phenomena associated with implants’ rupture (Ramião N. A. G. et al., 2017). Fatigue tests were then performed on virgin implants. The test data pointed out that (at least) some silicone shell ruptures are caused by cyclic loading (Ramião N. G. et al., 2017). Moreover, the shell thickness had significant variations, evidencing a heterogeneous structure when compared to other brands (Ramião N. A. G. et al., 2017). Biomaterial degradation was studied on virgin implant shells. Stiffening was observed induced by degradation (Martins et al., 2017). These phenomena, allied to tissue-tissue and tissue-implant friction and to external loads, may alter the implants’ performance and durability.

These findings had shown that new approaches for breast reconstruction are necessary to overcome the drawbacks, not only from the silicone implants but also from the autologous tissues. In research, regenerative medicine has become a real solution for breast reconstruction (Figure 1). The aim consists of the production of scaffolds or injectable hydrogels to promote adipose breast tissue regeneration (O’Halloran N. A. et al., 2018). Breast cancer affects mainly women in menopause and, in this phase, their breast lies on adipose tissue. Therefore, the main focus of research is regarding adipose tissue regeneration for breast reconstruction (Haddad et al., 2016).

Since the scaffolds are the ideal solution, their properties must be well documented. The material chosen, the biological and mechanical properties, as well as the morphology of the scaffold, must be studied and it should mimic the native breast tissue. Scaffolds must provide structural support for cells to attach, grow, migrate, and differentiate (Chan and Leong, 2008) as well as the required anatomical shape and sustain the mechanical forces usually applied in the defect site (Omidi et al., 2014; Griffin et al., 2016). Besides the required biocompatibility and biodegradability, the mechanocompatibility of the scaffold must also be considered (Janzekovic et al., 2020). The stiffness of the scaffold is extremely important since its structural integrity must be maintained while handling and despite the *in-vivo* physiologic forces. At the same time, it needs to be flexible enough to allow the in-growth of new tissue and vascularization. Moreover, its stiffness must mimic that of native tissue, since it will influence cells’ differentiation, tissue development, and tissue homeostasis (Engler et al., 2006; Horsnell and Baldock, 2016; O’Reilly and Kelly, 2016; O’Halloran N. et al., 2018).

The mechanical properties of human tissues, including the breast tissues, have been shown to have an important role in their function. Cells are sensitive to mechanical stimuli and, therefore they influence the normal behavior of the tissue, affecting not only healthy cells but also pathological cells. Through the transmembrane proteins, cells sense their microenvironment, regulating the physiological processes. It has been proven that the mechanical properties of the ECM, where cells are embedded, influence and it is influenced by the progression of neoplastic disease and that the rigidity of ECM affects the mobility of carcinoma cells (Cavo et al., 2016).

In literature, the mechanical properties of native breast tissues are not extensively reported, being inconsistent between studies. The non-standardized protocols for the *ex-vivo* experiments (different tests’ parameters and conditions) as well as the heterogeneity of the samples might contribute to this inconsistent data (Babarenda Gamage et al., 2017). Along with the heterogeneity of the samples, there are also the variables associated with the subject, such as age, weight, menopause, body mass index, etc., that influence the results as well. Therefore, a direct comparison between studies and an accurate validation of tissue-mimicking materials or scaffolds is difficult to achieve.

The state of the art for clinical breast reconstruction does not include regenerative medicine or the use of hydrogels. The *de facto* gold standard is the silicone implant, which has a finite lifespan and possible failure (Rohrich et al., 1998). Silicone implants have been studied (Ramião et al., 2017a,b; Ramião et al., 2017), and conclusions indicate that this solution has mechanical issues, which make them not fully reliable. To offer better solutions to women, regenerative medicine is taking important steps, being the implantable scaffolds and injectable materials the current focus of attention (O’Halloran N. et al., 2018).

In this context, an extensive review of breast tissue and its mechanical properties as well as the current solutions found in the literature for breast tissue regeneration will be presented in the following sections. Moreover, the approaches used in the literature regarding FE modeling for both breast tissues and breast scaffolds will be also detailed.

## 2 Breast tissue: Basic concepts and mechanical properties

**FIGURE 2:**
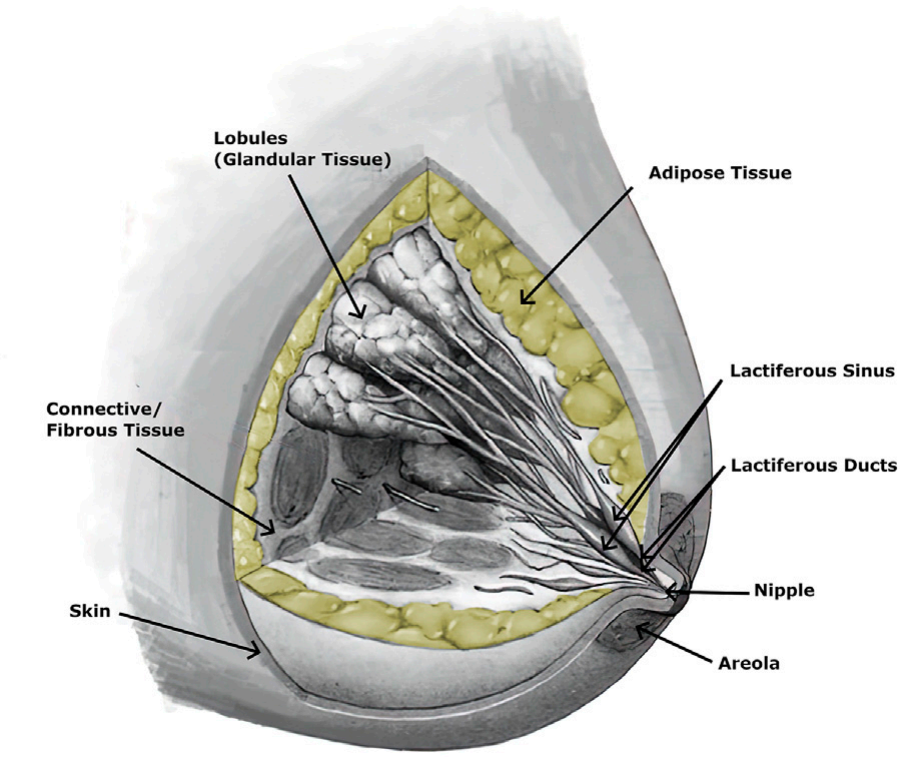
Anatomy of the breast, with adipose tissue colored yellow.

The breast is an important organ for women, being responsible for lactation (Babarenda Gamage et al., 2017). The breast has a heterogeneous structure composed of different tissues, such as adipose, glandular, and fibrous (Figure 2), in variable proportions between individuals, being dependent on age (Babarenda Gamage et al., 2017; Janzekovic et al., 2020). Each breast is organized in lobes of glands, called lobules, and contains the excretory ducts, which drain into the lactiferous sinus, radiating from the central nipple-areolar complex. Those lobes are embedded in fibrous and adipose tissues (Ramião et al., 2016), along with the nerves and blood and lymphatic vessels (Babarenda Gamage et al., 2017; García et al., 2018). To keep the shape and contour of the breast and hold it in place, there are the fibrous suspensory ligaments, named Cooper’s ligaments (Ramião et al., 2016; Babarenda Gamage et al., 2017).

Regarding breast tissues, besides morphology and structure, also the mechanical properties change along a woman’s life, due to factors such as age, menstrual cycle, pregnancy, menopause, lactation, etc. (Babarenda Gamage et al., 2017; García et al., 2018; Ng and Lin, 2019). An example is the stretching and weakening of the Cooper’s ligaments that are observed with aging (Ramião et al., 2016). There is evidence that fibroglandular tissue is 2 times stiffer during the menstrual cycle Lorenzen et al. (2003), being stiffer in the early follicular phase than in the luteal phase, but no significant differences were found in stiffness between adipose and glandular tissues (Li et al., 2015). Recently, Chen et al. (2019) compared the stiffness of fibrogladular tissues with the volumetric density of the whole breast as well as locally. The stiffness was 2.3 ± 0.8 kPa and it was not correlated with age, breast volume, whole breast percent density, or local percent density (ratio between fibroglandular tissue area and the whole area). However, breast density decreased with age. Moreover, pathologies also have a significant impact on the mechanical properties of breast tissues (Gefen and Dilmoney, 2007; Ramião et al., 2016), being shown that the intrinsic elasticity is a property that changes in a presence of a disease (Krouskop et al., 1998; Samani and Plewes, 2007; Ramião et al., 2016). The stiffness of the tissue depends on the micro- and macroscopic structure of the tissue, which is different under a pathological event.

Therefore, the mechanical behavior of the tissues plays an important role in the research of clinical applications, for example, cancer detection, surgical simulators, and tumor motion tracking during surgeries (Ramião et al., 2016). In clinical examination techniques, such as palpation or mammography, compression is applied on the breast to detect lesions, which are proven to be stiffer than normal tissues. Hence, it becomes very important to study the breast tissues under compression (Ramião et al., 2016), using *in-vivo*, and *ex-vivo* experimental techniques to mechanically characterize them.

The mechanical properties of soft tissues, such as breast tissue, are generally represented in the elastic and viscous domains (Fung, 1993), which, when combined, control the deformation of the tissue (Shiina, 2013). The available information regarding the hyperelastic mechanical behavior of the breast tissues is scarce and researchers have been focused on the measurement of the elastic modulus (Ramião et al., 2016). There are three types of elastic modulus: tensile, shear, and volumetric elasticity, being named Young’s modulus, shear modulus, and bulk modulus, respectively. The Young’s modulus, E, corresponds to the ratio between the longitudinal deformation in the direction of the applied load (strain, *ϵ*) and the response to the applied longitudinal load (stress, *σ*); The shear modulus, G, relates the transverse strain and stress; The bulk modulus, K, describes the change in volume of the material to external stress (Manickam et al., 2014). Young’s modulus is the most common to quantify stiffness in tissues (Ramião et al., 2016) and it can be obtained through the slope of the stress-strain curve (classic elasticity theory - Hooke’s Law), considering only the elastic region (i.e., the region where linearity is assured).

In several studies, soft tissues have been assumed to be linear elastic, near incompressible, and isotropic (Wellman et al., 1999; Han et al., 2003; Samani et al., 2003). The incompressibility is due to the high fluid content (mainly water) of the tissues, which confer a Poisson’s ratio of 0.495 (Han et al., 2003; Ramião et al., 2016). The Poisson’s ratio measures the transversal deformation relative to the longitudinal direction of load application. Assuming these features, tissues can be mechanically characterized using only the Young’s modulus, which is independent on the geometry or boundary conditions and dependent only on the properties of the material (Krouskop et al., 1998; Griffin et al., 2016). This can be only applied in quasi-static compression conditions, which corresponds to a very low frequency excitation.

The following equation (Eq. 1) has been used in literature to calculate the Young’s modulus (*E*) of breast tissues (Krouskop et al., 1998; Matsumura et al., 2009; Umemoto et al., 2014). However, it is only valid for a semi-infinite medium.

$$E=\frac{2\left(1-\nu^{2}\right)qa}{w}$$ (1)

where *ν* is the Poisson’s ratio, *q* is the load density (force per unit area), *a* is the radius of the loaded area and *w* is the maximum displacement in the direction of the load.

**FIGURE 3:**
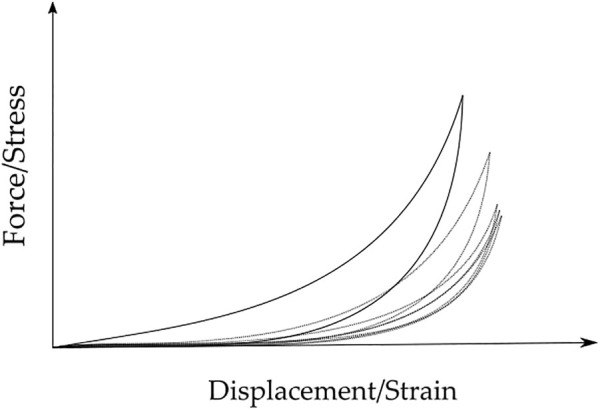
Typical Force/Stress vs. Displacement/Strain loading-unloading curves for breast tissue samples, over five cycles. Hysteresis decreases with the increase of cycles. Full line - first cycle; dotted lines - second to fifth cycles.

The linear elastic parameters give information, such as stiffness and deformability, at the macroscopic level, while the hyperelastic and viscoelastic parameters give information at the microscopic level (Omidi et al., 2014). The shear contact between the collagen fibers, proteoglycans, and elastin is the main cause of the viscoelastic behavior of the tissue (Ramião et al., 2016). In literature, breast tissues are often mechanically characterized considering the linear elastic Young’s moduli to quantify stiffness (Ramião et al., 2016), however, it is not suitable to characterize the tissues under large deformations (Omidi et al., 2014), which corresponds to the viscoelastic behavior. Due to this viscoelastic behavior, the tissues present different stress-strain curves during loading and unloading, being the loading-unloading cycles also different from each other (Fung, 1993) (Figure 3). This phenomenon is called hysteresis and it happens due to the energy dissipation caused by the shear stress, which results from the recovery of the tissue after elongation or contraction (Ramião et al., 2016).

The experimental tests to measure the mechanical properties of breast tissues can be divided into *in-vivo* (e.g., imaging techniques) or *ex-vivo* (e.g., compression and indentation tests).

### 2.1 *In vivo* experiments

The first diagnostic technique, performed either by the patients or by the doctors, is palpation. It is a qualitative method that allows the detection of large and superficial tumors. However, this technique is insensitive to small or deeper tumors in the breast. Moreover, it is highly dependent on the sensitivity and experience of the person who is performing the examination. Due to these factors, alternative or auxiliary methods are required (Ramião et al., 2016).

Imaging techniques are helpful tools in the diagnosis of abnormalities in the tissue, being simple to perform and non-invasive method. Elastography is a common technique that can help in the detection of large and superficial tumors (Ramião et al., 2016). It is a quantitative measure of the stiffness (e.g., elastic modulus) of the tissue under compression (Sinkus et al., 2000; Samani A. et al., 2001; McKnight et al., 2002; Van Houten et al., 2003; Srivastava et al., 2011). Compared to palpation, elastography has a higher level of sensitivity and specificity, being able to distinguish lesions from normal tissue as well as the type of lesion (malignant or benign), respectively (Wilson et al., 2000). Breast elastography also overcomes the limitations of current imaging diagnostic methods, such as mammography and ultrasonography. Mammography often gives false negative results in dense breasts and ultrasonography has poor specificity to distinguish between a malignant and benign lesion. Ultrasound (US) elastography is a non-invasive, more accurate, and highly specific technique that gives more information regarding the tissues, such as the elasticity (Goddi et al., 2012). Zhi et al. (2007) compared the three techniques, concluding that US elastography was the most specific for breast tissue analysis. However, the cancer detection diagnostics were more accurate when US elastography was combined with sonography.

**FIGURE 4:**
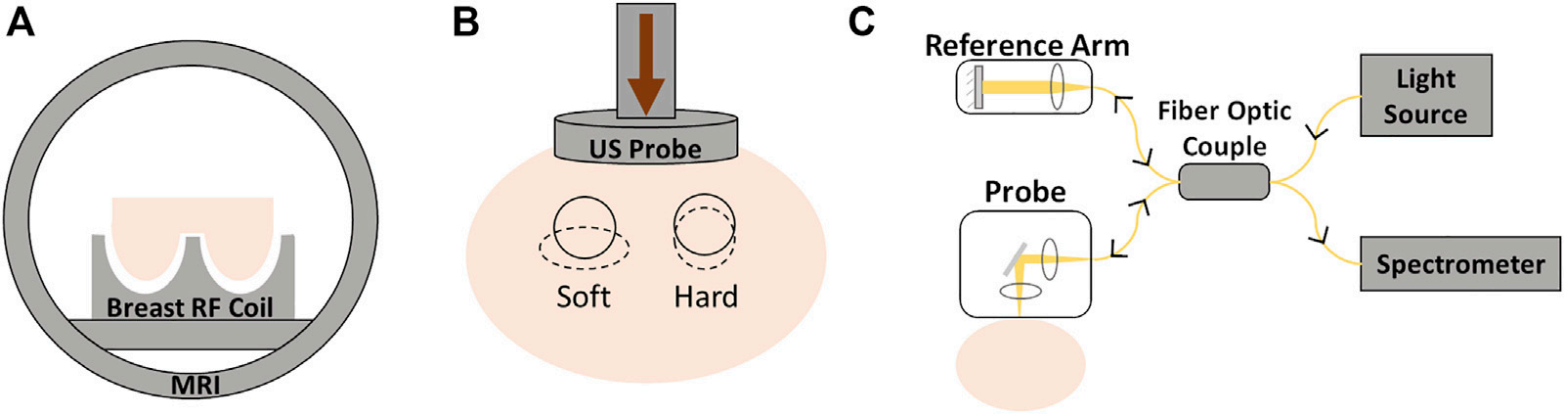
Different elastography techniques: **(A)** magnetic resonance elastography; **(B)** ultrasound elastography; **(C)** optical coherence elastography.

The imaging methods consist in applying stress or any controlled mechanical excitation on the tissue and measuring its response to that stimulus. That response will allow the determination of the parameters that reflect the mechanical properties (Mariappan et al., 2010). Depending on the mechanical stimulus applied to the tissue and the imaging modality to measure the response, elastography can be of different types: quasi-static or harmonic US elastography, magnetic resonance (MR) elastography, or optical coherence elastography (Ramião et al., 2016) (Figure 4).

A new elastography system based on a linear array transducer was introduced in 1993 by Céspedes et al. (1993). The output elastogram showed a well-defined black area, corresponding to a carcinoma, surrounded by white fat. The results showed that elastography is a capable imaging technique to estimate the elastic properties *in-vivo* with good resolution. It is able to detect deep and small local lesions in the tissue, which could be difficult to detect by palpation or other techniques.

Using MR elastography, Lawrence et al. (1998) investigated normal breast tissues and concluded that this technique is feasible, illuminating correctly with shear waves and characterizing properly the biomechanical properties of the tissues. They reported a stiffness of 2.45 ± 0.2 kPa for glandular tissue and 0.43 ± 0.07 kPa for adipose tissue. To study the potential of MR elastography to improve differentiation between benign and malignant tumors, Xydeas et al. (2005) studied the viscosity and elasticity of breast tissues. They found that the elasticity was higher for malignant tumors compared to benign tumors. Within benign lesions, the highest value of elasticity was from fibrocystic changes, followed by fibroadenoma and the surrounding tissue showed the lowest value. Chen et al. (2013) proposed a non-compressive breast MR elastography setup. For normal healthy women, they concluded that glandular tissues were stiffer than adipose tissues. Moreover, they analyzed the normal tissues of a diseased woman and her adipose and glandular tissues were stiffer than the ones of healthy women. For the diseased woman, invasive ductal carcinoma (IDC) was about 3 times stiffer than adipose tissue and 1.5 times stiffer than glandular tissue. The same conclusions were obtained by McKnight et al. (2002), which also used MR elastography in healthy and diseased women. The stiffness of breast carcinoma was 418% higher than the surrounding tissues. Only using healthy volunteers, Hawley et al. (2017) compared the stiffness of dense breasts (i.e., with a higher amount of fibroglandular tissue) and non-dense breasts. They concluded that the dense breasts had a mean stiffness of 0.92 kPa while the non-dense breasts had a value of 0.83 kPa. Comparing adipose with fibrogladular tissues, Van Houten et al. (2003) performed manual segmentation and proved that the adipose tissue was the softest. While Srivastava et al. (2011) included diseased tissues in the study and concluded that malignant and benign tissues were 4 and 2 times stiffer than normal tissues, respectively. Sinkus et al. (2000) obtained a stiffness of 2–3 times higher for carcinoma, compared to normal surrounding tissues. They reported as well that the carcinomas exhibited an anisotropic elasticity while surrounding tissues appeared to be isotropic. In 2005, Sinkus et al. (2005) studied the viscoelastic properties of different breast pathologies using elastography. They obtained the highest shear modulus and shear viscosity for breast cancers, in contrast with the surrounding tissue, which had the lowest values. Fibroadenoma and mastopathy had similar shear modulus but different shear viscosity.

Sayed et al. (2013) proposed a new diagnostic technique based on multicompression 3D US elastography. The results showed that the tumor was 6.3 times stiffer than the surrounding normal tissues, which was in good agreement with the result from the biopsy performed. Also using US elastography, Han et al. (2003) proved that the breast tissue had a viscoelastic behavior, more evident at large deformations. They observed the hysteresis effect between the loading and unloading curves, being stable after the second cycle. This evidence points to the need to apply preconditioning when investigating the mechanical properties of breast tissues.

Even though elastography is a reliable technique, there are some false negatives (Matsumura et al., 2009) that could be reduced if the test conditions, such as the adequate amount of initial stress, called precompression, were better understood. As an approach to this issue, Barr and Zhang Barr and Zhang (2012) tested four ranges of pre-compression during elastography: 0%–10%, 10%–25%, 25%–40%, and > 40%. They reported that when the compression was slight, the difference in stiffness between normal and tumor tissues was large, identifying more clearly the tumor region. However, when the precompression was higher, the stiffness of the normal tissue increased and the difference between those tissues and the tumor was smaller, which made the identification of tumor region more difficult. As a conclusion, the authors recommended that the clinical images should be obtained with a precompression of around 10% for more accurate results.

Presented as a supplementary material, Table 1 resumes the *in-vivo* results of the elastic properties of the breast tissue reported in the literature.

**TABLE 1:** *In-vivo* results of the elastic properties of breast tissues, reported in literature.

Experiment	Tissue types	Experimental protocol	Results/Conclusions	Authors
*In vivo*	Adipose and glandular tissues IDC (scirrhous carcinoma)	Elastography vs. Sonography	Healthy	Céspedes et al. (1993)
			Adipose tissue: soft (light) in the elastogram and hypoechoic in the sonogram	
			Glandular components: firm (dark) in the elastogram and hypoechoic in the sonogram	
			Patient	
			Well defined hard (black) area, within the soft (white) fat in the elastogram	
	Adipose and glandular tissues	MR elastography	E_glandular_ = 2.45 ± 0.2 kPa	Lawrence et al. (1998)
			E_adipose_ = 0.43 ± 0.07 kPa	
	Carcinoma	MR elastography	E_carcinoma_ = 3.5 kPa	Sinkus et al. (2000)
	Surrounding (benign) tissue		E_surrounding tissue_ = 0.5–1 kPa	
	Malignant lesions: invasive mucinous carcinoma IDC	MR elastography	E_cancer_ = 3.1 ± 0.7 kPa	Xydeas et al. (2005)
			E_fibroadenoma_ = 1.4 ± 0.5 kPa	
	Benign lesions: fibroadenoma fibrocystic changes		E_fibrocystic changes_ = 1.7 ± 0.8 kPa	
			E_surrounding tissue_ = 1.2 ± 0.2 kPa	
	Adipose and glandular tissues IDC	non-compressive MR elastography	Healthy	Chen et al. (2013)
			E_adipose_ = 0.33 kPa	
			E_glandular_ = 0.64 kPa	
			Patient	
			E_adipose_ = 0.41 ± 0.1 kPa	
			E_glandular_ = 0.90 ± 0.18 kPa E_IDC_ = 1.42 ± 0.17 kPa	
	Healthy women: Adipose Fibroglandular Patient with cancer: Invasive Carcinoma IDC ILC	MR elastography	Healthy: E_adipose_ = 3.3 ± 1.9 kPa	McKnight et al. (2002)
			E_fibroglandular_ = 7.5 ± 3.6 kPa	
			Patient	
			E_adipose_ = 4–16 kPa (mean 8 kPa)	
			E_tumors_ = 18–94 kPa (mean 33 kPa)	
	Adipose and fibroglandular tissues	MR elastography	E_adipose_ = 17.1067 ± 3.0283–23.5367 ± 4.0347 kPa	Van Houten et al. (2003)
			E_fibroglandular_ = 24.2871 ± 3.0939–30,2995 ± 3.4 kPa	
	Adipose tissue Fibroadenoma IDC	Optical coherence tomographic elastography	E_adipose_ = 4.17 ± 0.074 kPa	Srivastava et al. (2011)
			E_fibroadenoma_ = 9.03 ± 0.215 kPa E_IDC_ = 16.45 ± 1.103 kPa	
	Benign: Fibroadenomas Fibrocystic change Fibroadipose tissue Malignant: IDC ILC	Multi-compression	New estimated non-linear parameter benign = 0.163 ± 0.063	Sayed et al. (2013)
			malignant = 1.642 ± 0.261	
			Strain ratio (between soft and stiff tissues) benign = 2.135 ± 0.707	
		3D Ultrasound elastography	malignant = 4.21 ± 2.108	
			Relative mass volume benign = 0.848 ± 0.237	
			malignant = 2.18 ± 0.522	
	Normal breast tissues (healthy volunteer)	Ultrasound elastography	viscoelastic behavior, more evident at large deformations	Han et al. (2003)
			E = 1.832 kPa, for the maximum displacement of 20 mm	
	Dense and non-dense breast tissues (healthy volunteer)	MR elastography	E_dense_ = 0.92 kPa	Hawley et al. (2017)
			E_non-dense_ = 0.83 kPa	
	Adipose and fibroglandular tissues	Elastography precompression levels	Comparing malignant tissues with normal tissues	Barr and Zhang (2012)
	Fibroadenoma	A) 0–10∖%	A, B, and C: Difference in elasticity	
	Fibrocystic changes	B) 10–25∖%	D: the elasticity of both types of tissues was similar	
	Fat necrosis	C) 25–40∖%	Comparing benign tissues with normal tissues: only in level A, there were significant differences in elasticity	
	Malignancy	D) maior 40∖%	Young modulus (kPa) increases in all tissues as precompression increases	

### 2.2 *Ex-vivo* experiments

**FIGURE 5:**
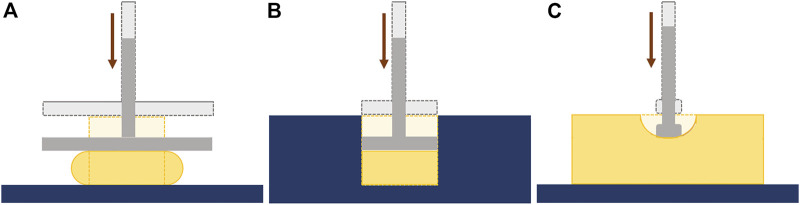
*Ex-vivo* mechanical tests: **(A)** Unconfined compression; **(B)** Confined compression; **(C)** Indentation.

**FIGURE 6:**
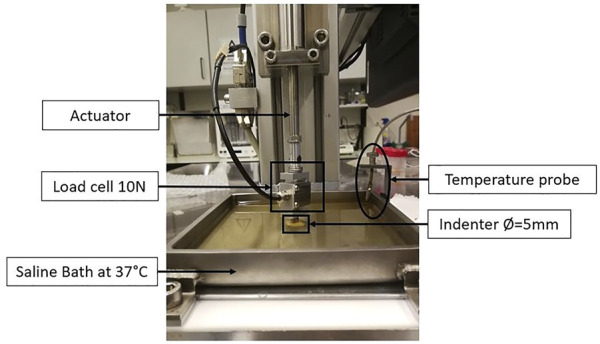
Example of an *ex-vivo* experimental setup to test human breast tissue samples, using a saline bath at 37°C, an indenter with 5 mm of diameter, and a load cell of 10N. Adapted from (Teixeira et al., 2023).

The influence of the test conditions, including the precompression, in the mechanical behavior of soft tissues, can also be studied by performing *ex-vivo* experimental tests, which include compression tests (confined or unconfined) and indentation tests (Figure 5) Griffin et al. (2016); Delaine-Smith et al. (2016). In these experiments, the displacement and the resulting force are measured and can be converted to strain and stress, respectively, and the Young’s modulus can be calculated. Some of the limitations of the compression tests are related to the geometrical irregularities and difficulties in cutting uniform samples without causing damage. Indentation can overcome this problem since little or no sample preparation is required (Delaine-Smith et al., 2016). A typical setup to analyze the mechanical properties of soft tissues, including breast tissues, is presented in the following figure (Figure 6).

It is known that the mechanical behavior of most soft tissues is non-linear, viscoelastic, and anisotropic. However, as a first approach, authors often assume that tissues are elastic, isotropic, and near incompressible in order to calculate the elastic modulus (Wellman et al., 1999; Han et al., 2003; Samani et al., 2003; Delaine-Smith et al., 2016). However, when large deformations are applied, these tissues exhibit viscoelastic behavior. This means that their mechanical response depends on the time elapsed since the load is applied (visco) and the initial state is recovered when the load is removed (elastic), indicating that there is no plastic deformation (e.g., damage) (Calvo-Gallego et al., 2020).

#### 2.2.1 Elastic properties

Sarvazyan et al. (1995), in 1995, tested normal breast tissues, fibroadenomas, and breast tumors. They concluded that fibroadenomas were 4 times stiffer than normal tissues and malignant tissues were 7 times stiffer than normal tissues. In 1998, Krouskop et al. (1998) studied different types of breast tissues such as adipose, glandular, fibrous, intraductal carcinoma, and infiltrating ductal carcinoma. They performed compression tests at three different frequencies (0.1, 1, and 4 Hz) and they applied a preload compression of 5% and 20%. The results showed that adipose tissue was the softest, being relatively constant over the loading range analyzed. Intraductal carcinoma *in situ* had an elastic modulus similar to adipose tissue at low strain, but at high strain, the modulus was larger than any normal tissue. Among all, IDC was the stiffest tissue. They also found out that the stiffness of breast tissues increases as precompression increases: for a 5% precompression the tumor tissue was 5 times stiffer than adipose tissue, while for 20% precompression the tumor was 25 times stiffer than adipose tissue. This dependency of tissue stiffness on preload confirms the non-linear behavior of tissues. Krouskop et al. (1998) also observed that tumor tissue, besides being the stiffest, had a higher non-linear increase in stiffness. This last conclusion was also obtained by Wellman et al. (1999), who performed indentation tests with a precompression of 2N over 10 cycles. They concluded that IDC was stiffer than normal tissues (adipose and fibroglandular), being this difference in stiffness higher at high strains.

Samani et al. (2003), in 2003, along with indentation tests, used FE analysis to obtain the Young’s modulus. These experiments confirmed the non-linearity of breast tissues and that the stiffness was sensitive to the amount of precompression. In Samani and Plewes (2007), the authors measured the Young’s modulus of tumors embedded in normal tissue and they observed that benign and malignant tumors were 5 and 10 times stiffer than normal breast tissues, respectively. Based on indentation tests, Samani et al. (2007) showed that normal tissues were the softest and the elastic modulus between adipose and fibroglandular tissues was similar. Compared to the normal tissues, fibroadenomas were 2 times stiffer, the fibrocystic disease was 6 times stiffer, and malignant tumors were 3–6 times stiffer. The IDC was the stiffest tissue, being 13 times stiffer than the normal tissues.

Using compression tests, Matsumura et al. (2009)measured the elasticity of breast tissues under the stress range usually applied in elastography (from 0.0 kPa to 1.2 kPa). They noticed a significant non-linearity in tissue elasticity and a difference in the Young’s modulus, depending on the compression status. Comparing normal tissues with carcinomas, the first ones were softer. However, ductal carcinoma *in situ* (DCIS) was only stiffer than normal tissues under slight stress. This behavior was changed around 1 kPa. Both normal tissues, adipose and glandular, showed a similar stress-strain curve, with higher non-linearity than lesions. IDC and mucinous carcinoma were the stiffer tissues. From the same group and using the same range of stress, Umemoto et al. (2014) concluded that the Young’s modulus increased in the following order: adipose, glandular, DCIS, and IDC. The difference in stiffness between normal tissues and lesions tended to gradually decrease as the stress applied increased (the stiffness of normal tissues increased to a point where they come close to or exceed that of malignant tissues), due to the non-linear properties. The rates of increase in elasticity of normal tissues are significantly larger than those of malignant tissues, showing a higher non-linearity in normal tissues.

Presented as supplementary material, Table 2 resumes the *ex-vivo* results of the elastic properties of breast tissue reported in the literature.

**TABLE 2:** *Ex-vivo* results of the elastic properties of breast tissues, reported in literature.

Experiment	Tissue types	Experimental protocol	Results/Conclusions	Authors
*Ex-vivo*	Adipose	Compression tests	Compression: 5∖%; 20∖%	Krouskop et al. (1998)
	Glandular	0.1, 1, and 4 Hz	E_adipose_ = 18 ± 7–22 ± 12; = 20 ± 8–24 ± 6	
	Fibrous DCIS	Compression of 5% and 20%	E_glandular_ = 28 ± 14–35 ± 14; = 48 ± 15–66 ± 17	
	IDC		E_fibrous_ = 96 ± 34–116 ± 28; = 218 ± 87–244 ± 85 E_DCIS_ = 22 ± 8–26 ± 5; 291 ± 67–307 ± 78 E_IDC_ = 93 ± 33–112 ± 43; 460 ± 178–558 ± 180	
	Adipose	Indentation tests 2N load for 10 cycles Strain rate: 50, 200, 1,000% and 2000%/s	Strain: from 0.01 to 0.15	Wellman et al. (1999)
	Glandular		E_adipose_ = 4.8 ± 2.5–17.4 ± 8.4	
	Phyllodes tumor		E_glandular_ = 17.5 ± 8.6–271.8 ± 167.7	
	Papilloma		E_phyllodes_ = 56.6–297.7	
	Lobular carcinoma		E_papilloma_ = 22.2 ± 5.8–537.8 ± 209.1 E_LC_ = 34.7–628.4	
	Fibroadenoma IDC		E_fibroadenoma_ = 45.5 ± 20.1–889.2 ± 205 E_IDC_ = 47.1 ± 19.8–1,366.5 ± 348.2 E_DCIS_ = 71.2–2,162.1	
	DCIS			
	Adipose	Indentation tests	E_adipose_ = 1.9 kPa	Samani et al. (2003)
	Fibroglandular	Preload = 0.5–2.0 g	E_fibroglandular_ = 1.8 kPa	
	High grade DC	0.5 mm and 0.02–0.1 Hz	E_carcinoma_ = 12.0 kPa	
		Preconditioning: 25 cycles + Test: 5 cycles		
	Fibroadenoma	Indentation tests	E_fibroadenoma_ = 11.42 ± 1.56 kPa E_DCIS_ = 14.15 ± 0.35 kPa E_ILC_ = 18.57 ± 0.85 kPa E_IDC_ = 22.55 ± 2.95 kPa	Samani and Plewes (2007)
	High grade DCIS	Preload = 0.01–0.03 N		
	Infiltrating LC	0.5 mm and 0.1 Hz		
	IDC	Preconditioning: 25 cycles + Test: 5 cycles		
	Adipose	Indentation tests Preload = 3.0 g 0.5 mm and 0.1 Hz Preconditioning: 25 cycles + Test: 5cycles	E_adipose_ = 3.25 ± 0.91	Samani et al. (2007)
	Fibroglandular		E_fibroglandular_ = 3.24 ± 0.61	
	Fibroadenomas		E_fibroadenoma_ = 6.41 ± 2.86	
	Low grade IDC		E_lowIDC_ = 10.40 ± 2.6 E_ILC_ = 15.62 ± 2.64 E_DCIS_ = 16.38 ± 1.55	
	ILC			
	DCIS			
	Fibrocystic disease		E_fibrocystic_ = 17.11 ± 7.35	
	Intermediate grade IDC		E_intIDC_ = 19.99 ± 4.2	
	High grade IDC		E_highIDC_ = 42.52 ± 12.47 E_IMC_ = 20.21	
	IMC			
	Fat necrosis		E_necrosis_ = 4.45	
	Adipose	Indentation test Up to 30% strain at 1 mm/min (50% for fat or gland)	E_adipose_ = 0.69 ± 0.19–19.08 ± 4.99	Umemoto et al. (2014)
	Glandular		E_glandular_ = 0.73 ± 0.18–16.99 ± 4.92	
	IDP		E_IDP_ = 3.13 ± 1.74–12.30 ± 4.29	
	DCIS		E_DCIS_ = 5.25 ± 0.46–16.15 ± 4.24	
	Invasive carcinomas		E_invasive_ = 13.82 ± 9.60–30.50 ± 11.46	
	IDC, ILC, MC, Metaplastic carcinoma			
	Adipose	Indentation tests Up to 30% strain at 1 mm/min (50% for fat or gland)	E_adipose_ = 0.7 ± 0.2–17.3 ± 4.8	Matsumura et al. (2009)
	Glandular		E_glandular_ = 0.8 ± 0.2–15.4 ± 3.9	
	DCIS		E_DCIS_ = 3.4 ± 1.3–15.6 ± 2.0	
	MCIDC		E_mucinous_ = 9.2 ± 1.7–18.9 ± 2.3	
			E_IDC_ = 11.5 ± 8.4–27.0 ± 9.2	
	human DAT (breast)	Indentation tests 0.5mm and 0.1 Hz Preconditioning: 25 cycles + Test: 5 cycles	E_DAT_ = 3.460 ± 1.210 kPa	Omidi et al. (2014)

#### 2.2.2 Viscoelastic properties

Focusing on the hyperelastic behavior, Samani and Plewes (2004) performed indentation tests and successfully measured the adipose and fibroglandular breast tissue hyperelastic parameters, using an inverse technique based on FE modeling. A similar protocol was used by Dempsey et al. (2021), in which adipose, fibrogladular, and mixed tissues were tested *via* indentation to estimate the hyperelastic properties using 4 models. They concluded that the three types of tissues were statistically similar, which corroborates the use of a homogeneous model for large strain simulation. Also focused on the viscoelastic behavior, Calvo-Gallego et al. (2020) compared the properties of adipose breast tissue with abdominal adipose tissue. They performed uniaxial compression relaxation tests and fitted a mechanical model to the experimental curves. They found out that adipose breast tissue has unique mechanical properties. They observed that the differences between breast and abdominal adipose tissue were related to the viscous constants and not to the elastic ones. This means that under static loading, their behavior is similar, but under dynamic loading their behavior is different. Looking only for the mechanical behavior of IDC, Mojra and Hooman (2021) performed ramp-relaxation tests and estimated the viscoelastic properties by using the Maxwell model. They concluded that the relaxation time decreased with the strain level, which indicates that more time is needed, at higher strains, for the relaxation of IDC samples.

Omidi et al. (2014) had the goal to compare the linear elastic and hyperelastic properties of human decellularized adipose tissue (DAT) and normal breast adipose tissue. Through indentation tests, the force-displacement data was acquired, and, using inverse FE analysis, the elastic and hyperelastic parameters were calculated. They concluded that DAT from the breast showed a deformability similar to native normal tissue, with a Young’s modulus of 3.460 ± 1.210 kPa, which is close to the values of normal adipose breast tissue in literature (3.250 ± 0.910 kPa (Samani et al., 2007)). Moreover, they found out that DAT from different regions of the body presented little intrinsic non-linearity, with no significant differences between them. Haddad et al. (2016) also studied the biomechanics of DAT of breast and subcutaneous abdominal depots using the same approach. They also concluded that both DATs have similar deformation to normal breast tissue under the same loading conditions.

Focusing on adipose tissue, Sun et al. (2021c) used dynamic compression and simple shear loading tests to compare human abdominal and porcine adipose tissues. The tissue was found to be non-linear and could be modeled as a one-term Ogden hyperelastic material. They observed that the porcine adipose tissue was stiffer than human adipose tissue. Also in 2021, the same group (Sun et al., 2021b), characterized the hyper-viscoelastic properties of human abdominal subcutaneous adipose tissue, by performing the same *ex-vivo* tests. They applied multiple ramps and hold tests to evaluate the quasilinear viscoelasticity and they showed that the non-linear, viscoelastic, and direction-dependent responses could be obtained with the Ogden hyperelastic model.

Presented as supplementary material, Table 3 resumes the results of the hyper-viscoelastic properties of breast tissue reported in the literature.

**TABLE 3:** Results of the hyper-viscoelastic properties of breast tissues, reported in the literature.

Experiment	Tissue types	Experimental protocol	Results/Conclusions	Authors
*Ex vivo*	Adipose Fibroglandular	Indentation tests Preload = 0.5 g 1.0mm and 0.1 Hz Preconditioning: 25 cycles + Test: 5 cycles + Inverse FEM Polynomial strain energy function (N = 2)	Hyperelastic parameters	Samani and Plewes (2004)
			(*C*_10_, *C*_01_, *C*_11_, *C*_20_, *C*_02_) x 10^−4^ Nmm^-2^	
			Adipose (3.1 ± 0.3, 3.0 ± 0.2, 22.5 ± 3, 38.0 ± 6, 47.2 ± 7)	
			Fibroglandular (3.3 ± 0.4, 2.8 ± 0.3, 44.9 ± 8, 77.2 ± 11, 94.5 ± 13)	
	Adipose (breast and abdominal)	Compression tests Preconditioning: 20 cycles: 10% at 1 Hz Test: 50% at 50%/s + hold for 15 min + Internal variable viscoelastic model elastic part (first-order Ogden - *μ* and *α*) + viscous part (superposition of exponentially decreasing functions - *β*_1_, *β*_2_, *β*_3_, *β*_4_, *β*_5_)	*Breast deep tissue*	Calvo-Gallego et al. (2020)
			*μ* = 0.058, *α* = 8.875	
			*β*_1_ = 67.286, *β*_2_ = 20.520, *β*_3_ = 5.584, *β*_4_ = 3.160, *β*_5_ = 2.824	
			*Breast superficial tissue*	
			*μ* = 0.057, *α* = 7.949	
			*β*_1_ = 65.934, *β*_2_ = 16.891, *β*_3_ = 3.446, *β*_4_ = 1.861, *β*_5_ = 1.623	
	Adipose Fibroglandular Mixed Tissues	Indentation tests 0.5–1.0mm and 0.1 Hz Preconditioning: 25 cycles + Test: 5 cycles + FEM models 3rd order Ogden, Veronda-Westman, 2nd order Polynomial and Yeoh models	No statistically significant differences among stress distributions	Dempsey et al. (2021)
	IDC	Ramp-relaxation tests 2, 4, 6% strain at 0.1s^-1^ + hold 180s + FEM model Maxwell model	Long-term shear moduli = 0.31–17.03 kPa	Mojra and Hooman (2021)
			Instantaneous shear moduli = 6.03–55.13 kPa	
	Human DAT (breast and abdominal)	Indentation tests	Mean difference (9 points) of displacements between	Haddad et al. (2016)
		Preload = 0.1 g	“normal breast” and post-mastectomy(1)/post-lumpectomy(2)	
		1.5 mm and 0.1 Hz	breast reconstructed using various DAT materials for a	
		Preconditioning: 20 cycles + Test: 5 cycles	prone-to-supine(3)/prone-to-upright(4) body position change	
		+		
		FEM models	Yeoh: (1,3) 5.5 mm; (1,4) 5.4 mm; (2,3) −4.0 mm; (2,4) −0.6 mm	
		Yeoh and Ogden models	Ogden: (1,3) 0.8 mm; (1,4) 0.8 mm; (2,3) −5.4 mm; (2,4) −0.7 mm	
	Human DAT (breast)	Indentation tests	E_DAT_ = 3.460 ± 1.210 kPa	Omidi et al. (2014)
		Preload = 0.1 g	*First-order polynomial:* (C01, C10) x 10^−2^	
		0.5 mm and 0.1 Hz	3.917 ± 2.653, 9.997 ± 4.671	
		Preconditioning: 20 cycles + Test: 5 cycles	*Yeoh:* (C10, C20, C30)	
		+	0.1554 ± 3.728 × 10^−2^, (1.575 ± 0.440)x10^−2^, (8.820 ± 2.901)x10^−8^	
		FEM models	*Ogden:* (*μ*, *α*)	
		First-order polynomial, Yeoh, Ogden,	0.3306 ± 8.109 × 10^−2^, 3.780 ± 0.5431	
		and Arruda–Boyce models	*Arruda–Boyce:* (*μ*, *λ*)	
			0.1813 ± 0.1026, 1.028 ± 0.1655	
*In vivo*	Cancer cases Fibroadenoma Mastopathy cases	MR Elastography	*Shear modulus, kpa*	Sinkus et al. (2005)
			Breast cancer = 2.9 ± 0.3; Fibroadenoma = 1.3 ± 0.7	
			Mastopathy = 1.2 ± 0.4, Surrounding tissue = 0.87 ± 0.15	
			*Shear Viscosity, Pa.s*	
			Breast cancer = 2.4 ± 1.7, Fibroadenoma = 2.1 ± 1.4	
			Mastopathy = 0.8 ± 0.3, Surrounding tissue = 0.55 ± 0.12	

### 2.3 Extracellular matrix

The mechanical properties of human tissues, including the breast tissues, have been shown to have an important role in their function. ECM determines the cell fate and biological activities of cells (Ghajar and Bissell, 2008; Keller et al., 2021; Tamayo-Angorrilla et al., 2022), which are sensitive to mechanical stimuli. Therefore, ECM mechanics has an important role in cell behavior and shape and function of tissues, either healthy or diseased (Cavo et al., 2016; Mierke, 2021). It is known that changes in ECM mechanics and composition are involved in cancer progression and metastasis (Tamayo-Angorrilla et al., 2022). During carcinogenesis, the stiffness of ECM is continuously changing due to ECM remodeling, which involves activation of cancer-associated fibroblasts and excessive extracellular collagen deposition, crosslink, and fibrosis (Keller et al., 2021; Deng et al., 2022; Tamayo-Angorrilla et al., 2022). Collagens are the most abundant structural proteins in ECM, which provide support, mediate drug resistance, promote tumor progression and aggressive cell transformation, and affect the mechanics of the tissue (Tamayo-Angorrilla et al., 2022) Therefore, different content and density of collagens have a significant impact on tissue stiffness and its variation (Deng et al., 2022), which will be sensed by cancer cells (Mierke, 2021) and, hence, influence cancer progression (Cavo et al., 2016) The morphology, proliferation capacity and invasive ability of cancer cells change as a result of the mechanotransduction process of the physical signals send by the ECM stiffness (Deng et al., 2022). Moreover, ECM stiffness also has an impact on the response to cancer treatments (Tamayo-Angorrilla et al., 2022), by controlling the sensitivity of the tumor cells (Deng et al., 2022).

Acerbi et al. (2015) observed the collagen accumulation in breast cancer as well as the linearization and thickening of the interstitial collagen. The linearization was more evident in invasive tumors where the ECM stiffness was higher. Also, the stiffer and more heterogeneous tumors were the more invasive and aggressive ones. Also, Keller et al. (2021) concluded that invasive tumors are stiffer. They studied the structural and mechanical properties of human normal breast and IDC tissue, observing that IDC was stiffer than normal tissues and the ECM of IDC was stiffer than the ECM of normal tissues. They obtained a similar Young’s modulus for normal breast tissue and the respective ECM, however when comparing IDC tissue to its ECM, the ECM had a significantly higher Young’s modulus.

### 2.4 Breast tissue: Final remarks

As an overall conclusion, the stress-strain curves of breast human tissues present a non-linear (exponential) behavior, being adipose tissue the one that presents a curve closer to the linear behavior. Malignant cancer has the highest Young’s modulus, while normal breast tissues are the softest. The studies showed that as more invasive the tumor, the greater the elastic modulus (e.g., the high-grade invasive cancer is the stiffest, compared to other tumors (Samani et al., 2007)). The increase in stiffness with pathology results from a modification of the structure of the normal tissue components, such as elastin, collagen, and proteoglycans, which corresponds to an increase in the elastic modulus of the tissue (Ramião et al., 2016). Pathologies not only present a higher stiffness but also have a higher non-linear increase in stiffness compared to normal tissues (Krouskop et al., 1998). At small strains, the elastic modulus is similar between tissues, however, at large strains, cancerous tissues are much stiffer than normal tissues. Therefore, to distinguish between malignant and benign cancer it should be considered data from large strains, which implies the measurement of non-linear parameters (Manickam et al., 2014).

Apart from normal breast tissues, Cooper’s ligaments are very important (particularly for younger women) due to their function of holding the breast in place and giving it support. However, their mechanical behavior is very hard to investigate, probably due to the difficulty of extracting them from a specimen as well as in experimental handling since they are extremely fragile. Therefore, besides the surgical approaches to collecting these ligaments, there is a need to develop techniques to mechanically test them. This knowledge can contribute to a better understanding of the mechanics of the breast and, hence, investigate plastic surgery techniques and alternative approaches for breast reconstruction. In the literature, there is only one study concerning the mechanical properties of Cooper’s ligaments, however, the samples came from a cadaver. Briot et al. (2020) performed uniaxial tensile fracture tests on these ligaments and they obtained a Young’s modulus equal to 5.8 ± 4.2 MPa, a rupture strain as 8.6% ± 4.2% and a rupture stress of 1.9 ± 2.5 MPa. The results were fitted with a hyperelastic constitutive equation, the Neo-Hookean model.

## 3 Breast tissue scaffolds

As an alternative to implants and prostheses, regenerative medicine and tissue engineering have been growing trends in research. Those techniques try to replace or regenerate the damaged or diseased organ or tissue by combining cells from the patient with biomaterials (O’Halloran N. A. et al., 2018). Therefore, even though the clinical gold standard for breast reconstruction is implant-based, significant research improvements have been proposed using scaffolds and injectable hydrogels.

### 3.1 Fabrication considerations

**FIGURE 7:**
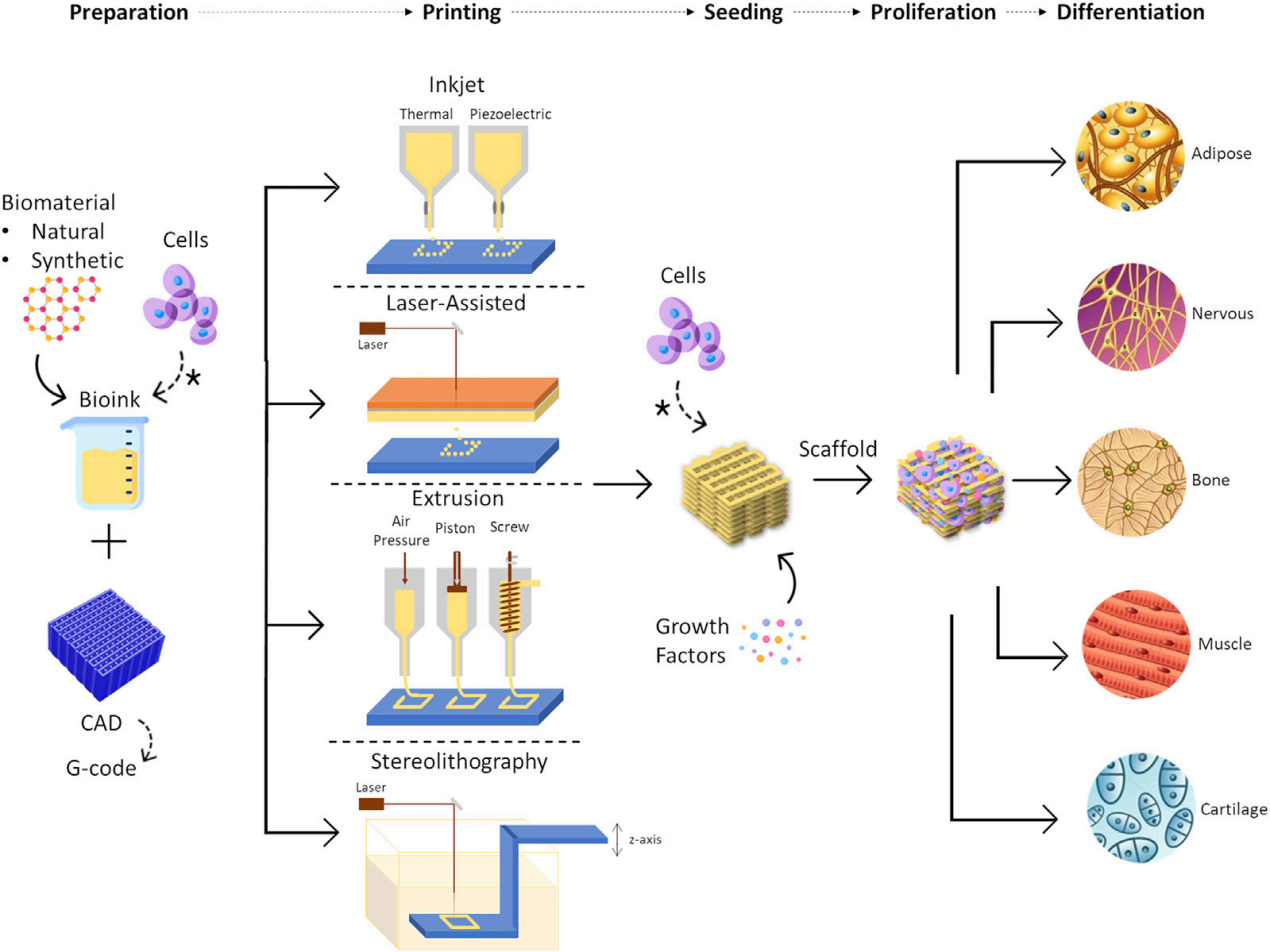
Representation of 3D bioprinting steps, including the different printing techniques that can be used, such as inkjet printing, laser-assisted printing, extrusion printing, and stereolithography. Some examples of tissues that can be regenerated using this approach are represented as the outcome of the process. * means cells can be placed at the beginning of the process, in solution with the biomaterial or they can be seeded into the scaffold after the printing process.

A scaffold should allow the production of native-like tissue, with similar bio, physical and chemical properties (O’Halloran N. et al., 2018; Donnely et al., 2020). They should provide 3D structural integrity, contain cell-specific signaling cues, and be non-toxic (Chae et al., 2018). Techniques to produce scaffolds include 3D printing, which is currently a promising and growing approach since idealized tissues/organs can be developed, combining cells with biomaterials into scaffolds (Mandrycky et al., 2016) to accurately mimic the native tissue (O’Halloran N. et al., 2018) (Figure 7). In 3D printing, a 3D construct is developed in a layer-by-layer fashion from a computer-aided design, being possible to produce custom designs with complex internal morphology and perform controlled material extrusion to achieve the desired biomechanical properties (Chae et al., 2018; Mohseni et al., 2018). There are different 3D printing techniques, such as inkjet, extrusion, laser-assisted, and stereolithography printing (Figure 7) (Chae et al., 2018; Cleversey et al., 2019).

The requirements for scaffold production depend on the 3D printing technique and the target tissue. For instance, if it is inkjet printing, the bioink needs to have low viscosity and low thermal conductivity to avoid clogging and heat damage, respectively. On the other hand, in extrusion printing, the materials can have a higher viscosity but the mechanical properties become more important since the degree of cell damage increase (Bishop et al., 2017). The 3D printing technique chosen will be dependent on the tissue of interest and the scalability required. For example, for soft and large tissues, such as breast tissue, the most adequate technique is the one that has a fine resolution to the degree of vasculature size, with high speed and low costs (Cleversey et al., 2019).

When a scaffold is being developed, the material properties, blueprint, architecture, and cells must be taken into account (Rocco et al., 2016; O’Halloran N. et al., 2018). Through the porosity and pattern of the scaffold, the functional features, mechanical behavior, and mass transport properties can be tailored (Rocco et al., 2016). Moreover, the material printability and structural integrity after printing can be influenced by the viscosity of the material, surface tension during printing, cross-linking process, gelation kinetics, degradation rates, and cell encapsulation densities (Cleversey et al., 2019).

The degradation rate is crucial since the scaffold should remain intact during the time required to form the new tissue but should degrade at a rate that allows its substitution by the new ECM (O’Halloran N. et al., 2018). Degradation may occur *via* different mechanisms: by hydrolysis, typically in synthetic polymers, by enzymatic cleavage, common in natural polymers, or by dissolution. The degradation rate can be manipulated by changing the cross-linking density, which is easily achieved in synthetic hydrogels. It is paramount that the degradation by-products are nontoxic, cause limited inflammation, and do not activate the immune response (O’Halloran N. A. et al., 2018). It is a concern the possibility of harmful by-products resulting from the degradation of synthetic polymers (O’Halloran N. et al., 2018), which can cause a change in the pH of the environment or inflammation.

Besides biodegradability, the mechanical behavior of the scaffold is also important. It should mimic the properties of the native tissue to be replaced since if it is too rigid it can cause mechanical irritation and scar tissue formation but if it is too soft the structure can collapse (Omidi et al., 2014). Soft matrices might induce neurogenic phenotype, while rigid matrices resembling bone result in osteogenesis, and matrices that mimic muscle encourage myogenic differentiation (O’Halloran N. A. et al., 2018). At a macroscopic level, its stiffness and rigidity must be adequate to support the forces that the tissue usually suffers and must provide stability for the new tissue to grow. On the other hand, at a microscopic level, it must provide attachment sites, mechanical cues, and growth factors to cells to ensure their growth and proliferation (O’Halloran N. A. et al., 2018). The scaffold’s mechanical and chemical properties and its mineralization influence the proliferation and differentiation into a cell’s lineage (Chae et al., 2018; Donnely et al., 2020). By studying adenocarcinomas, Cavo et al. (2016) showed that the cell viability decreased with the increase of elasticity of the alginate substrate and that cell proliferation was highest in the softest hydrogel. Besides cell proliferation and differentiation, the mechanical properties will also influence the formation of the vasculature, which will influence the transport of nutrients and waste. Natural polymers have enhanced vascularization over synthetic polymers, however, it can be improved by adding growth factors (e.g., vascular endothelial growth factor and fibroblast growth factor) or cells (e.g., ADSCs) (O’Halloran N. A. et al., 2018).

To build a successful scaffold, porosity is another relevant parameter to consider. The presence of microchannels will allow vascular infiltration and growth, facilitating oxygen and nutrient diffusion (O’Halloran N. A. et al., 2018). The pores’ interconnectivity is crucial for cell migration, proliferation, and differentiation. Moreover, the pore size is also important since it must be able to accommodate cells of different sizes. In the particular case of adipose tissue regeneration, those cells are ADSCs (10 *μ*m), differentiated adipocytes (100 *μ*m), and mature adipose tissue lobules (300–500 *μ*m) (Chae et al., 2018). The pore size must be adequate to simultaneously support angiogenesis and adipogenesis (Mohseni et al., 2018).

**FIGURE 8:**
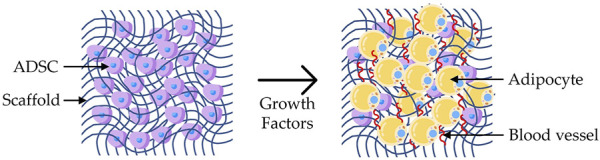
Adipogenesis: representation of a scaffold seeded with ADSCs and cultured with growth factors, leading to the differentiation into adipocytes and growth of blood vessels.

In 3D bioprinting, the most common cells are mesenchymal stem cells (MSCs). Since they are capable of producing any tissue, the microenvironment must be tightly regulated to produce the desired outcome (Chae et al., 2018). Regarding adipose tissue regeneration, the most successful approach is using ADSCs seeded in an appropriate scaffold (Tytgat et al., 2019b), with growth factors and endothelial precursor cells in co-culture (Chae et al., 2018) (Figure 8). ADSCs are adult MSCs found in several tissues, especially in adipose tissue. Therefore, they can be isolated by liposuction (Kokai et al., 2014; Tytgat et al., 2019b), from the stromal vascular fraction (Visscher et al., 2017). They can self-renew and have multipotent differentiation into osteoblasts, chondrocytes, myocytes, neurocytes, vascular endothelial cells, and adipocytes (Kokai et al., 2014; Tsuji, 2014; Chae et al., 2018; Cleversey et al., 2019). Therefore, ADSCs are ideal for adipogenesis (O’Halloran N. A. et al., 2018) and angiogenesis (Donnely et al., 2020). When they are seeded into soft scaffolds (e.g., human-derived DAT scaffold) that mimic the stiffness of native adipose tissues (i.e. 2–4 kPa), adipogenic differentiation is promoted, even in the absence of exogenous adipogenic growth factors (O’Halloran et al., 2018a; b; Chae et al., 2018). The main limitation of these cells is the potential contribution to breast cancer recurrence (O’Halloran et al., 2017; Visscher et al., 2017; Chae et al., 2018; Donnely et al., 2020), due to secreted adipokines (O’Halloran et al., 2017), which is not fully understood due to the lack of long-term studies to conclude on the overall safety of those cells (Cleversey et al., 2019).

### 3.2 Biomaterial

The scaffold biomaterial is key when the goal is to regenerate a specific type of tissue (O’Halloran N. A. et al., 2018) since the properties of this biomaterial (mechanical and chemical) will affect cell adhesion, proliferation, and differentiation (Aliabouzar et al., 2018; O’Halloran N. et al., 2018; Cleversey et al., 2019; Donnely et al., 2020; Zhou et al., 2020). To select the best bioink, the stiffness of the tissue and its vasculature network must be considered. Moreover, the physical properties of the bioink (structure strength, resolution, and shape) must be compatible with the printing technique (Cleversey et al., 2019). Regarding the scaffold biomaterial, it can be a natural or a synthetic polymer.

#### 3.2.1 Natural polymers

Natural or biological polymers are materials that exist in the body (Mohseni et al., 2018) and/or have molecular properties similar to those of native ECM (O’Halloran N. A. et al., 2018). Their major advantage are their good cell interaction (good biocompatibility (O’Halloran N. et al., 2018; Mohseni et al., 2018)), supporting cell viability and growth, their biodegradability (Carletti et al., 2011; Ratheesh et al., 2017), and their similar structure to native ECM (Cleversey et al., 2019; Donnely et al., 2020), however, they have poor mechanical strength (Chae et al., 2018) and rapid degradation in the presence of bodily fluids or culture media (O’Halloran N. A. et al., 2018). For example, gelatin and alginate have poor shape sustainability and print resolution, therefore they form very soft gels at physiologic temperatures. Crosslinking, to introduce new functional groups, or produce a composite with other biomaterials, has successfully improved their mechanical properties (Bishop et al., 2017; O’Halloran et al., 2018a,b). Some examples of natural polymers are collagen, agarose, hyaluronic acid, alginate, silk, chitosan, gelatin, fibrin, and decellularized ECM (O’Halloran N. A. et al., 2018; Chae et al., 2018; Cleversey et al., 2019; Donnely et al., 2020).

The interest in hydrogels is growing fast, showing great potential in tissue engineering, drug delivery applications, and as a coating for medical devices, being highly biocompatible due to their large water content. Hydrogels have been widely studied for their potential in adipose tissue regeneration, due to their biocompatibility, low inflammation, and suitable/tunable mechanical properties (O’Halloran N. A. et al., 2018), mimicking accurately the natural ECM (Zigon-Branc et al., 2019). These materials can be characterized in the time and frequency domains, exhibiting time-dependent mechanical behavior due to their viscoelasticity, and time-dependent deformation mechanism due to their fluid flow (Oyen, 2014), which is an important parameter for the development of a material in regenerative medicine (Bootsma et al., 2017). Hydrogels are also commonly used in 3D printing to mimic and regenerate human tissues, such as skin, vessels, neural tissue, cartilage, adipose tissue, and skeletal muscle, among others (Mandrycky et al., 2016).

Hydrogels are a hydrophilic porous network, capable of absorption and retention of large quantities of water or fluids. The crosslinks, ionic or covalent bonds, between the polymer chains define their structural properties and their physical properties, such as swelling ratios, elastic modulus, and degradation, are influenced by the type of crosslinking (physical or chemical) (O’Halloran N. A. et al., 2018). Hydrogels allow the incorporation of live cells within the scaffolds and can be altered in order to deliver growth factors and mechanical signals to those cells. To accurately mimic the ECM, hydrogels have been modified by adding bioactive molecules such as cell-adhesive peptides, enzyme-sensitive peptides, and growth-factor binding (O’Halloran N. A. et al., 2018).

Functionalized gelatins (e.g., gelatin methacrylated (GelMA), thiolated gelatin, and gelatin-norbornene and/or methacrylated carrageenan) are some examples of hydrogels that have been studied for the regeneration of human adipose tissue. After printed, these matrices were seeded with ADSCs and their regenerative capacity was evaluated. Ovsianikov et al. (2010) used GelMA and concluded that after photo-polymerization, the hydrogel preserved its enzymatic degradation capability. Moreover, the developed scaffolds, using two-photon polymerization, showed to support primary ADSCs adhesion, proliferation, and differentiation. Also using GelMA (Van Hoorick et al., 2015), developed self-supporting low-density porous gelatin hydrogels, using indirect additive manufacturing fused deposition modeling (FDM). The indirect 3D printed scaffolds provided an interconnecting porous network and cell attachment to the scaffold with a successful low mortality rate. Using the same approach, Markovic et al. (2015) developed polylactic acid (PLA) scaffolds by FDM and used a solution of GelMA in a cell culture medium containing a photoinitiator as a precursor of the hydrogel. The results indicated that the scaffolds supported preosteoblast cells’ survival and proliferation over the experience time. To compare the indirect with direct extrusion-based 3D printing techniques, Damme et al. (2020) used GelMA as the hydrogel for the scaffold and PLA for the molds (in indirect printing). No significant differences were found in the physical-chemical properties of the scaffolds from both techniques, being the indirect method more beneficial for low-viscosity materials.

Producing a 1 mm sheet structure, Salamon et al. (2014) reported that gelatin-based hydrogels showed a promotive effect on chondrogenic differentiation of MSCs *in-vitro*. Zigon-Branc et al. (2019) analyzed the influence of the stiffness of gelatin-based hydrogels in the proliferation and differentiation of microspheroids formed from telomerase-immortalized human ADSCs. Confocal microscopy indicated that all the tested hydrogels supported cell viability. While in the softer hydrogels cells started outgrowing and interconnecting within a few days, in stiffer hydrogels their protrusion was slower. It also confirmed the presence of calcium deposits in osteogenically stimulated samples in the two softer hydrogels.

As an alternative to the current GelMA hydrogels, Tytgat et al. (2019a) used thiolene photo-click crosslinkable gelatin hydrogel (norbornene-functionalized gelatin combined with thiolated gelatin). The results showed that the extrusion-based 3D printed scaffolds were able to mimic the physicochemical properties of the ECM of adipose tissue in terms of swelling properties, mechanical strength, and *in-vivo* biodegradability. The seeded ADSCs remained viable for up to 14 days and they were able to proliferate and differentiate into the adipogenic lineage. The same group used the same gelatin modification at different concentrations and compared it with GelMA with different degrees of substitution (Damme et al., 2021). A sheet with 1 mm height was produced and a physicochemical characterization and a biological evaluation were performed. The results showed that the hydrogels had similar properties compared to GelMA, exhibiting a mechanical behavior close to adipose tissue. The hydrogels presented a higher differentiation of ADSCs into the adipogenic lineage, compared to GelMA. Producing a different gelatin modification, (Tytgat et al., 2019b), printed two types of 3D extrusion-based scaffolds: only with GelMA and a blend of GelMA and methacrylated k-carrageenan. Both remained stable over time, were able to absorb large amounts of water, and exhibited mechanical properties similar to native adipose tissue (2 kPa). ADSCs were seeded in both scaffolds and a similar cell viability was obtained. Furthermore, the cells differentiated into the adipogenic lineage, with higher differentiation potential in the GelMA scaffold than in the hydrogel blend scaffold. The ideal pore size for adipogenic differentiation should range between 500 *μ*m and 1,000 *μ*m.

From a different group but also using gelatin, Sutrisno et al. (2021) produced scaffolds with black phosphorus nanosheets to kill breast cancer cells and induce adipose tissue reconstruction. The scaffolds were produced by lyophilizing a mixture of gelatin with black phosphorus nanosheets and ice particles. It was found that the scaffolds with a high amount of black phosphorus nanosheets were able to kill the cancer cells *in-vitro* and *in-vivo* under laser irradiation. Also, the scaffolds were cultured with human MSCs and they promoted lipid oil droplet formation and upregulated the expression of adipogenesis-related genes.

Regarding adipose tissue regeneration, besides gelatin-based hydrogels, hyaluronic acid, alginate, or DAT have been also studied for this purpose, being suitable to culture with ADSCs (Van Nieuwenhove et al., 2017).

##### 3.2.1.1 Biological polymers: ECM components

The interest in decellularized ECM is increasing. They cause minimal immunologic and inflammatory responses and mimic accurately the native tissue microenvironment, since the structure is preserved, acting as a natural template for the remodeling of regenerated tissue. In the decellularization process, the cellular components of the ECM are removed but the biological properties remain intact. However, the yield of this process is small, which makes it not reliable for large reconstructions. Collagen type I is the main component of ECM and the possibility of altering the mechanical properties of the scaffold makes it promising to adipose tissue engineering (O’Halloran et al., 2018b,a). Using collagen, Puls et al. (2021)developed a regenerative tissue filler to be applied in a liquid state during breast-conserving surgeries, and then it forms *in situ* a fibrillar collagen scaffold. It was observed that these scaffolds induced breast tissue regeneration, such as adipose and glandular tissues, and no foreign body response was observed. Moreover, it has the advantage to conform to patient-specific defects.

With a four-chamber slide as mold, Sokol et al. (2016) produced 3D scaffolds made of extracellular proteins and carbohydrates present in human breast tissue and were cultured in a serum-free medium and seeded with primary human breast epithelial cells isolated from the patient. Those cells rapidly self-organized in the absence of stromal cells and within 2 weeks expanded to form mature mammary tissues, containing luminal, basal, and stem cells in the correct topological orientation and exhibiting the complex ductal and lobular morphologies observed in the human breast.

Using DAT with natural polymers, Cheung et al. (2014) evaluated the response of human ADSCs encapsulated in an injectable scaffold containing DAT (as a cell-supportive matrix) and methacrylated glycol chitosan or methacrylated chondroitin sulfate (as delivery vehicles). This method showed high seeding efficiency and uniformity. The DAT enhanced the ADSCs viability, retention, and adipogenesis within the gels. Comparing the hydrogels, methacrylated chondroitin sulfate had a better performance *in-vitro* and *in-vivo*.

Also, commercial acellular dermal matrices (ADM) have been studied for breast reconstruction. Carruthers et al. (2015) evaluated the biochemical composition and structure of AlloDerm Regenerative Tissue Matrix and AlloMax Surgical Graft in a porcine model of a tissue expander. They found out that the AlloMax had quicker incorporation in the host tissue and higher cell infiltration, fewer foreign body giant cells, and faster remodeling. Maxwell and Gabriel (2016) proposed a combination of an ADM with an expander or implant followed by fat grafting. The expander is placed subpectorally with a sheet of ADM at the bottom. Then, another piece of ADM is placed at the top of the expander followed by autologous fat injections. The authors applied this concept in over 500 reconstructions and obtained good outcomes and low complication rates.

In addition to being used in breast tissue regeneration, biological polymers have also been used for the reconstruction of the nipple-areolar complex. When a mastectomy is performed, the nipple-areolar complex is lost as well, being its regeneration of great importance for women. Therefore, nipple-areolar complex reconstruction is a common procedure for the whole breast reconstruction. Areola reconstruction has been accomplished with autologous skin grafts, tattooing, and ADM such as AlloDerm. To recreate the nipple projection, local skin flaps have been used. However, this flap-based approach has poor long-term cosmetic outcomes (Visscher et al., 2017). With augmented-flap reconstruction, this issue might be overcome by introducing a central core of biomaterial into the flap to increase the structural integrity (Khoo et al., 2019). Tissue engineering is a promising technique in this context as well, but few studies have been exploring this issue. Creating autologous tissue-engineered cartilage in the shape of a human nipple, Cao et al. (1998) used pluronic F-127 as an injectable scaffold and seeded it with autologous auricular chondrocytes. They used porcine models to recreate nipples, which were tattooed after 3 weeks to create the appearance of a human nipple-areolar complex. After 10 weeks, they obtained nodules with similar size, shape, and texture to human nipples. Pashos et al. (2017) successfully characterized a decellularized nipple-areolar complex obtained from non-human primate rhesus macaque and compared it with native tissues. Moreover, the resultant biological scaffold was cultured with MSCs and a high degree of bioactivity was obtained. More recently, from the same group, Caronna et al. (2021) implanted those acellular grafts into two rhesus macaque non-human primates and successfully assessed the safety and host-mediated re-cellularization over 6 weeks, using the native nipples as control.

#### 3.2.2 Synthetic polymers

Synthetic polymers are widely used in tissue engineering as their mechanical properties, degradation rate, hydrophobicity, and biological features can be highly controlled in a well-organized fashion tailored to specific applications and functions (O’Halloran N. A. et al., 2018). Synthetic polymers have higher mechanical stability over time than natural polymers (Donnely et al., 2020), and growth factors and ECM components are easily added. These polymers are flexible and their chemical and physical features are controllable, being possible to achieve optimal porosity, surface characteristics, and degradation rate, with low variability between batches (Mohseni et al., 2018). Due to a lack of peptides and binding sites, their main drawback is their poor biocompatibility (Mohseni et al., 2018), which requires modifications on the surface through the design or chemical modification, for example, incorporating bioactive domains such as RGD (Arg–Gly–Asp) sequence, which will allow cell attachment and proliferation (O’Halloran N. A. et al., 2018; Chae et al., 2018; Cleversey et al., 2019).

Some examples of synthetic polymers are polycaprolactone (PCL), PLA, poly(lactic-co-glycolic acid), polyethylene glycol, and Pluronic F127 (O’Halloran N. A. et al., 2018; Chae et al., 2018; Donnely et al., 2020). Zhou et al. (2020) created breast scaffolds of polyurethane to evaluate the influence of microstructure on the mechanical properties. The scaffolds, produced using FDM, presented the same porosity but different architectures: N5S4, N9S8, N7S6, and N4S6 (crystal lattices of diamond, tungsten, sodium chloride, and copper, respectively). They concluded that N5S4 was the softest scaffold with a stiffness similar to that of breast tissue, higher adipose survival, higher vascularization, and milder fibrosis. The deformation and the stress distribution of each scaffold as well as the influence of the unit cell architectures on the mechanical properties were also investigated by FE analysis.

Also with a focus on scaffold microstructure but using a stereolithography-based printer, Aliabouzar et al. (2018) used polyethylene glycol diacrylate (PEGDA) to produce 3D printed porous scaffolds, with different microstructures (solid, hexagonal, and square pores), and they showed that porosity and pore geometry are crucial for the properties of scaffolds. Increasing the porosity, the elastic moduli and sound speed decreased and attenuation was highest for scaffolds with hexagonal pores. Moreover, porous scaffolds, especially with square pores, had a higher cell attachment and growth of human MSCs. PEGDA scaffolds showed properties similar to soft tissues, being suitable for their regeneration.

Focusing on the geometry as well, Mohseni et al. (2019) printed, using FDM, 3D produced 3D printed patient-specific scaffolds made of PCL and composed by two different structures, for medium to large-volume regeneration for breast reconstruction. The external structure provides biomechanical stability while the internal structure provides adequate porosity and interconnectivity to guide tissue formation. This methodology allows tuning the architecture of the external structure and its stiffness, optimizing the regions with a higher risk of stress concentration or crack propagation. In terms of internal structure, it allows the application of a variety of geometrical features, with a gradient of porosity to minimize fat movement and avoid fat leaking, after fat injection through an additional channel structure. Moreover, a FE model was used to analyze the effects of architecture on the mechanical properties of the external structure, by performing uniaxial compression tests with a rigid flat platen and applying load to specific regions with different mesh densities. The proposed design enables to customize the architecture of the external structure in terms of mechanical properties and to apply different geometrical features in the internal structure. This combination allows producing patient-specific scaffolds, which can be further combined with fat injection.

With the same polymer, Meng et al. (2020) developed flexible scaffolds with tissue-specific geometry and mechanical properties for soft tissue engineering, using selective laser sintering and sinusoidal filament networks. This technique allows to tune the elastic modulus and increases the flexibility of the scaffolds. In 2021, the same group (Meng et al., 2021) used the same PCL scaffolds to design helical architectures and to study their large deformation response. Through experimental tests and FE model, they found out that under large deformations the scaffolds with more uniform deformation patterns and flexible properties were the ones with interlaced helical filament networks. The proposed FE model was shown to be able to predict the mechanical responses of patient-specific breast scaffolds in the implantation site with the simulated and experimental volume changes of the breast cavity.

One of the problems related to the combination of scaffolds with ADSCs is to make the regeneration of large volumes of adipose tissue feasible for clinical purposes (Janzekovic et al., 2020). An approach to overcome this problem was proposed by Chhaya et al. (2016), using a combination of a extrusion-based 3D printed, biodegradable and patient-specific scaffold made of medical grade PCL, with a delayed fat injection. The scaffold was first implanted to promote vascularization, through the formation of a blood clot that consists of a fibrin network together with a growth-factor cocktail of fibronectin, vitronectin, and thrombospondin, which stimulates a strong angiogenic response and induces highly organized connective tissue to penetrate into the affected region. After 14 days, the angiogenic response was at its peak and autologous fat was, at that time, injected into the scaffold. With this approach, the area of new adipose tissue was similar to native breast tissue, and the highest when compared with lipoaspiration or scaffold implantation alone.

Combining scaffolds with fat injection, Rocco et al. (2019) also developed 3D PCL scaffolds, using FDM. They printed different designs and performed compression tests and rheological analyses to characterize them. Additive manufacture The goal is to implant the scaffold which would be subcutaneously, and in 2-3 sessions they would fill it with autologous fat tissue. This hybrid reconstruction, as the authors called it, has the advantage, over silicone implants, to be an option for women undergoing post-mastectomy radiotherapy, since it will not be influenced by irradiation.

Also to address the problem of adipose tissue regeneration in large-volume defects, Jain et al. (2020) developed an extrusion-based 3D printed scaffold made of medical-grade copolymer coated with polydopamine. They investigated how the printing influences the molar mass of the polymer (which decreased over printing time) and the mechanical properties of the scaffold (which changes over printing time due to gradual degradation); then different printing designs were explored and, the surface functionalization was assessed. They conducted *in-vitro* cell studies with human ADSCs and concluded that the polydopamine increased cells attachment, proliferation, and adipogenic differentiation.

Using PCL and FDM technique, Griffin et al. (2020) produced scaffolds with different porosities and pore architectures. They coated the scaffolds with similar properties to human breast tissue but they used a coating of platelet-rich plasma to enhance the adipocyte proliferation of 3T3-L1 adipocytes. The scaffold most similar to human breast tissue in terms of compressive properties was the one with 40% porosity and square pores. It was also shown that the platelet-rich plasma coating enhanced adipocyte formation, tissue integration, and vessel formation *in-vivo* using a mice model. Cheng et al. (2021) also carried out an *in-vivo* study using a porcine model, where they implanted a 3D printed (extrusion-based) PCL scaffold for breast tissue engineering. They were able to grow clinically relevant volumes of soft tissue over a long-term period, validating their model for tissue regeneration strategies. More recently, Jwa et al. (2022) printed spherical 3D PCL scaffolds. They produced and implanted in rats three types of PCL scaffolds: 1) only PCL, 2) PCL and collagen, and 3) PCL with rat breast tissue fragment. The PCL scaffold had high compressive strength and showed morphology recovery properties. After 6 months of implantation, the scaffold with collagen increased adipose and fibrous tissue regeneration, contrary to what happened to the scaffold with breast tissue fragment. Nevertheless, there was no difference in the inflammatory response.

### 3.3 Breast tissue scaffolds: Final remarks

To overcome the limitations found in the current clinical approaches for breast reconstruction, promising solutions in tissue engineering have been investigated. Researchers have been studying not only possible solutions for breast tissue regeneration but also ways to reconstruct the nipple-areola complex. For women, this also has an important impact on their appearance and physiological state. In this sense, scaffolds have been the focus of study, as a support for cells to adhere, migrate, proliferate, and differentiate in order to grow new native-like tissue. 3D printing techniques have been used to produce the scaffolds since customized geometries and architectures can be printed. The ideal scaffold must have good biodegradability, high mechanical strength, dimensional stability, high processability, high porosity, and interconnectivity, be permeable to oxygen, nutrients, and metabolic wastes (Mohseni et al., 2018; Donnely et al., 2020), have a high surface-to-volume ratio, and good biocompatibility to prevent long-term immune reactions (Rocco et al., 2016; Ratheesh et al., 2017; O’Halloran N. et al., 2018; Janzekovic et al., 2020). Different materials have been applied to produce scaffolds and combined with cells, mainly ADSCs. Both natural and synthetic materials have their advantages and disadvantages, therefore in literature, there is a variety of biomaterials used. Gelatin-based hydrogels, especially functionalized gelatin, polyurethane, PEGDA, PCL, DAT, and collagen are some successful examples of materials already investigated for this purpose.

Table 4 resumes the 3D printed scaffolds described in the literature, identifying the 3D printing technique, the material used, and the conclusions achieved by the authors.

**TABLE 4:** 3D printed scaffolds for breast tissue regeneration, reported in the literature.

Fabrication process	Type of material	Material	Cells (*in-vitro*) or Model (*in-vivo*)	**Conclusions**	**Authors**
Two-Photon Polymerization	Natural	GelMA	ADSCs	Cells adhesion, proliferation and differentiation	Ovsianikov et al. (2010)
Extrusion-based (indirect 3D printing)	Natural	GelMA	Human foreskin Fibroblasts	Interconnected porous network	Van Hoorick et al. (2015)
				Cell attachment with low mortality rate	
Extrusion-based	Natural	Thiolene photo-click crosslinkable gelatin hydrogel	ADSCs	Thiolene photo-click crosslinkable gelatin hydrogel	Tytgat et al. (2019a)
Extrusion-based	Natural	GelMA + methacrylated k-carrageenan	ADSCs	Mechanical properties similar to native adipose tissue	Tytgat et al. (2019b)
				Cells differentiated into the adipogenic lineage,	
				Ideal pore size: 500–1000 *μ*m	
Extrusion-based (direct vs. indirect 3D printing)	Natural	GelMA	-	No differences in physical-chemical properties	Damme et al. (2020)
				Indirect printing better for low-viscosity materials	
Stereolithography-based	Synthetic	PEGDA	MSCs	Increasing the porosity, the elastic moduli	Aliabouzar et al. (2018)
				and sound speed decreased	
				Higher attenuation for hexagonal pores scaffolds	
				Higher cell attachment and growth with square pores	
Extrusion-based	Synthetic	PCL with fat injection	-	*External structure:* biomechanical stability	Mohseni et al. (2019)
				*Internal structure:* adequate porosity and interconnectivity to guide tissue formation	
Extrusion-based	Synthetic	PCL with fat injection	-	It will not be influenced by irradiation Maintain the breast shape and natural consistency	Rocco et al. (2019)
Extrusion-based	Synthetic	PCL with fat injection	-	Area of new adipose tissue similar to native breast tissue	Chhaya et al. (2016)
				Better results compared to lipoaspiration or scaffold implantation alone	
Extrusion-based	Synthetic	Poly(L-lactide-co-trimethylene carbonate) + polydopamine coating	ADSCs	Polydopamine increased cells attachment, proliferation, and adipogenic differentiation	Jain et al. (2020)
Selective laser sintering	Synthetic	PCL	-	Interlaced helical filament networks: more uniform deformation patterns and flexible properties	Meng et al. (2020, 2021)
				FE model: mechanical responses in the implantation site	
Extrusion-based	Synthetic	Polyurethane	Rat model	N5S4 architecture was the softest scaffold (stiffness similar to breast tissue), with higher adipose survival, higher vascularization, and milder fibrosis	Zhou et al. (2020)
Extrusion-based	Synthetic	PCL + platelet-rich plasma coating	3T3-L1 adipocytes	Best scaffold for breast tissue: 40% porosity and square pores Coating enhanced adipocyte formation, tissue integration, and vessel formation	Griffin et al. (2020)
			Mice model		
Extrusion-based	Synthetic	PCL	Porcine model	Grow clinically relevant volumes of soft tissue over a long-term period	Cheng et al. (2021)
Extrusion-based	Synthetic + Natural	PCL, PCL with collagen,	Rat model	*PCL scaffold:* high compressive strength and morphology recovery properties	Jwa et al. (2022)
		PCL with breast tissue fragment		*PCL with collagen scaffold:* after 6 months increased adipose and fibrous tissue regeneration	
				No difference in the inflammatory response between PCL combinations	

## 4 Finite element analysis

### 4.1 Finite element models: Breast tissue

To improve the knowledge about the mechanical behavior of the breast, some authors have been using FE models. The numerical models are based on biomechanics and geometry, and each model is characterized by the specific material properties of the breast (Babarenda Gamage et al., 2017) and the boundary conditions (Ramião et al., 2016; García et al., 2018). The boundary conditions, as well as the internal structure of the breast, can be obtained from medical images, such as MR images (Babarenda Gamage et al., 2017). The adipose and fibroglandular tissues, the pectoral muscle, and the tumors can be directly segmented from the medical images, however small structures, such as Cooper’s ligaments, are very difficult to identify. The thickness of the skin is normally obtained from experimental data available in the literature and the vessels and nerves are excluded due to their reduced mechanical contribution to the breast as a whole (Babarenda Gamage et al., 2017).

**FIGURE 9:**
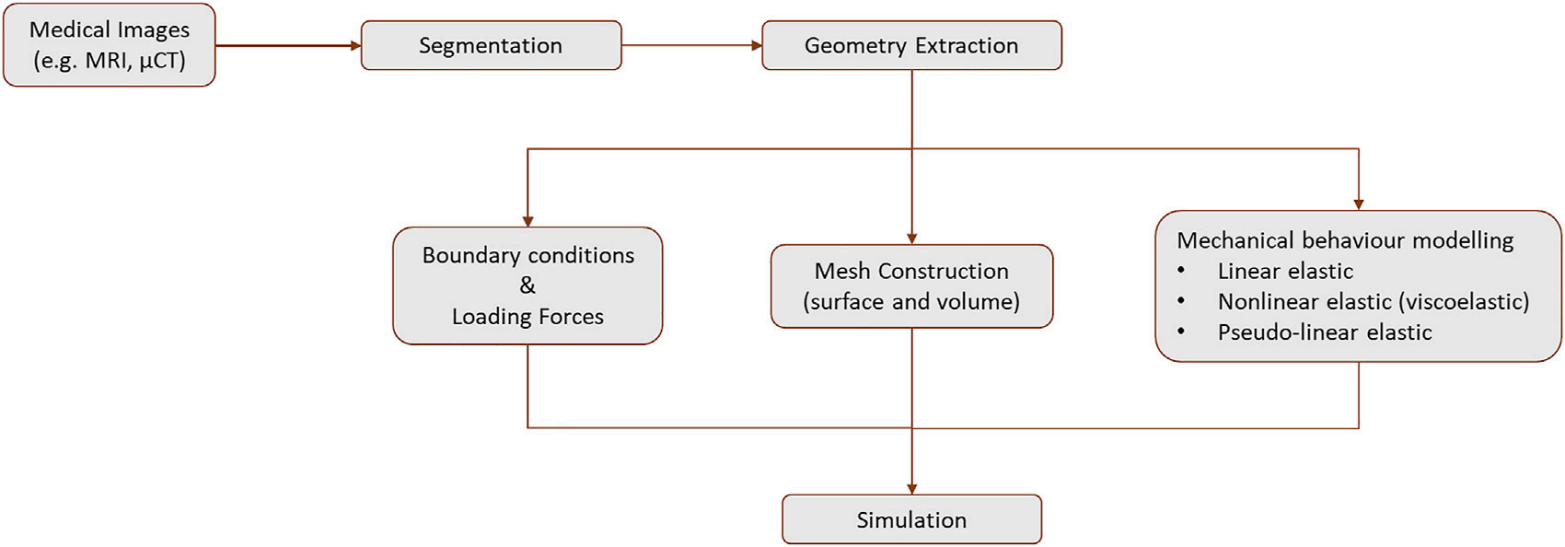
Workflow of the steps to reach a finite element model.

**FIGURE 10:**
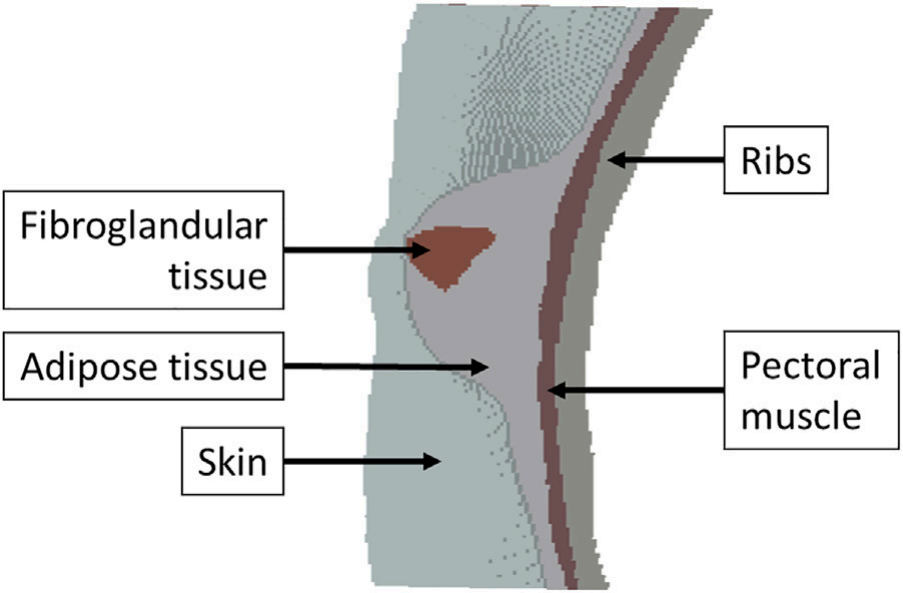
Cross section view of the FE model of a human breast. Adapted from (Areias et al., 2022)

**FIGURE 11:**
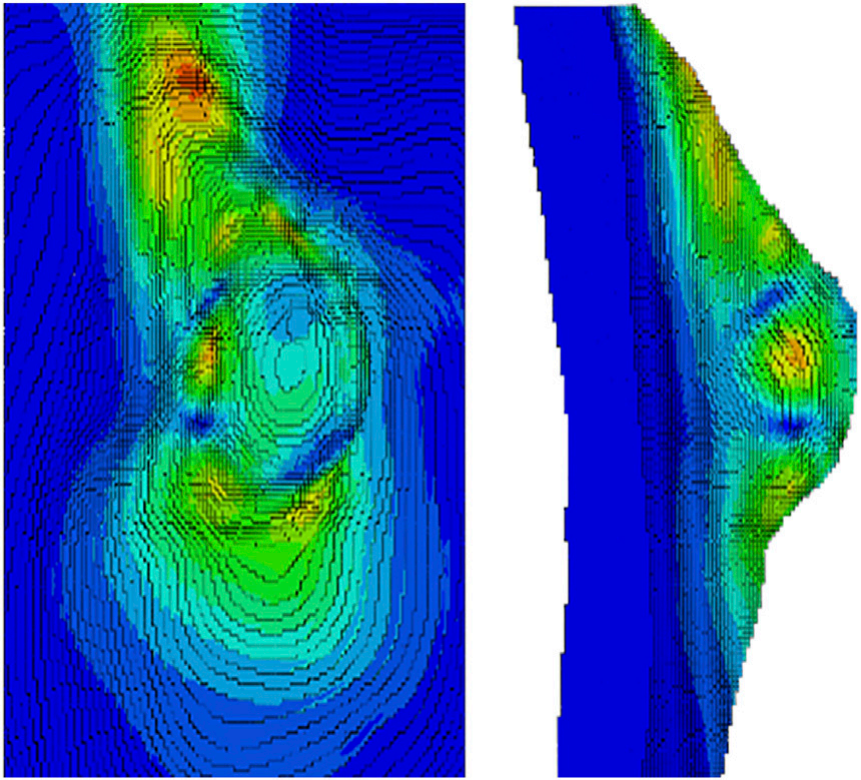
Normal breast tissue simulation - the numerical result of the natural frequency of shape mode 10. Adapted from (Areias et al., 2022)

Figure 9 provides a schematic overview of the steps required to create a FE model of the breast. The first step to design a model is the geometry extraction from the medical images by segmentation and mesh construction (surface and volume). Then, the mechanical behavior can be modeled as linear elastic, non-linear elastic (viscoelastic), or pseudo-linear elastic. The loading forces are applied and the boundary conditions are defined. The Neo-Hookean, Mooney-Rivlin, Yeoh, and Arruda-Boyce models are the most common material models to model the breast (García et al., 2018). Following these steps, an example of a FE model to simulate the human breast is presented in Figures 10, 11. This model was created using MR images to investigate the static and dynamic behavior, such as the natural frequency of a normal breast (Areias et al., 2022). The accuracy of the models to predict the *in-vivo* behavior of the breast is strongly dependent on the mechanical properties defined for each tissue. In addition, the patient-specific complex morphology of the breast, its hyperelastic mechanical behavior, and the difficulties of measuring the mechanical properties of the different types of tissues contribute to the challenge of modeling the breast of each patient (Ramião et al., 2016).

Breast models are a helpful tool in the diagnosis of diseases since it is easier to map and combine information from different imaging techniques, being possible, for example, to predict the exact location of a tumor (Babarenda Gamage et al., 2017). An example is the study of Unlu et al. (2010), where it was developed and tested a FE method for the registration of MR to positron emission tomography non-rigid breast images. This method matches MR with positron emission tomography images and can be fully automated by including markers detection and matching and mesh generation. They obtained a deformed FE mesh that reasonably approximates the non-rigid deformation of the breast tissue between the MR and positron emission tomography scans.

Each medical image is acquired under different conditions, including the position of the patient which can be prone (e.g., MR images) or supine (e.g., computed tomography (CT) images). Tracking features within the breast between these two positions or in a standing position, under the effect of gravity, is highly important for clinical applications (Babarenda Gamage et al., 2017; Danch-Wierzchowska et al., 2017, 2018), such as postural deformation analysis (Na et al., 2019), surgical planning (Samani et al., 2003; Na et al., 2019) or implant performance analysis (Dong et al., 2016; Myung et al., 2019; Na et al., 2019).

Danch-Wierzchowska et al. (2018) created a two-dimensional model, assuming the breast as isotropic, linear elastic, and homogeneous, and they studied the breast deformation from prone to supine position using MR images. Even though the results were acceptable for small and medium breasts, to represent a real situation a 3D object should be used instead of one by one image and the parameters need to be tuned for bigger breasts. Na et al. (2019) created 3D breast shapes, by using the geometrical information of fat and fibroglandular tissues obtained from MR images, taken in the prone position. They applied a Neo-Hookean material model and used FE analysis to build a 3D model of the breast in. The breast was deformed to correspond to its natural standing posture shape, by imposing gravity. The proposed method may increase the accuracy of surgery, by taking part in its preparation. Chen et al. (2019) also used the Neo-Hookean model to simulate the zero-gravity state of the breast, using US and MR images. They start with the prone position, which was pulled down by gravity, until the supine position, which corresponded to an inversion of the gravity state. The breast was meshed into a large number of tetrahedrons that had various sizes, to be able to modify the geometry but keep the topology. Each tetrahedron, corresponding to adipose or fibroglandular tissue, was modeled as isotropic and homogeneous. The surface adjacent to the chest wall was fixed, as a boundary condition. With this model, they tried to correlate breast stiffness (fibrogladular tissue in the central area of the breast) measured by US and breast density measured by MR imaging, however, the results showed no correlation between these properties, indicating that the stiffness is not only related to the amount of fibroglandular tissue.

Regarding the silicon breast implants, the deformation of the breast under gravity with two different types of implants in standing and lying positions was evaluated by Dong et al. (2016), using MR images. They concluded that the deformation caused by gravity was smaller in the standing position, being firmer and maintaining the shape. Myung et al. (2019) developed a two-dimensional model of the breast with the implant to track the changes and stress variation after breast implantation, using the Mooney-Rivlin model. In terms of external changes, the dimension of the implant and the top-point were not correlated to any change. However, related to internal changes, the stress applied to the lower thorax was higher for implants with a lower top-point. Also, the maximum stress was higher for dynamic analysis than for static analysis.

Besides studying the influence of gravity, mainly between patients’ positions, also the impact of compression is highly important. The mechanical properties of the breast are, as mentioned before, a key element to diagnose pathologies, which leads to diagnostic techniques, such as mammography or elastography, based on compression (Danch-Wierzchowska et al., 2017). Therefore, modeling the breast under compression, mimicking what happens in a mammography exam, was the topic of research for Sturgeon et al. (2016) and Liu et al. (2017). They used, respectively, CT data and MR images to build the model and applied the Mooney-Rivlin and the Neo-Hookean constitutive models, respectively. Sturgeon et al. (2016) generated mammograms by simulating gravity and compression in breast phantoms. The results were realistic, which indicates that this FE method can be used to simulate imaging data. Liu et al. (2017) studied the compressed breast thickness and concluded that it has a strong correlation with breast volume but a weak correlation with glandularity. The same was concluded by (Chang et al., 2022), who developed a breast model, using MR images, to optimize the breast compression used in digital mammography. The obtained compressed breast thickness was in good agreement with the clinical measure and, as expected, it decreased as the compression force increased. The information suggests a subject-specific compression force considering image quality and patient comfort. Focusing more on the stress of the breast and of a lesion, Axelsson et al. (2022) simulated the mammographic compression using two different sizes of breast models (linear elastic material) and inserted a spherical lesion, which varied in location and stiffness. They obtained stress values of 6.2–6.5 kPa for the breast and 7.8–11.4 kPa for the lesion. These results are in accordance with clinical measurements, which validates the proposed model for the evaluation and optimization of mechanical imaging screening techniques.

Samani a. et al. (2001) presented a biomechanical model of the breast, using a FE formulation and emphasizing the deformation under breast imaging procedures. The FE mesh was produced using MR breast images, obtaining patient-specific models. They simulated adipose and fibroglandular tissues using eight nodded hexahedral elements with hyperelastic properties and they simulated skin tissue with four nodded hyperelastic membrane elements. The validation of this model was made through a FE mesh of an agarose phantom, using MR images. Based on the assigned elasticity parameters, a numerical experiment was performed with the FE meshes and good qualitative results were obtained. Also using, MR images, Azar et al. (2001) constructed a deformable FE model of the breast, by modeling the mechanical properties of the tissue with a non-linear model. The purpose of this model was to predict the position of the tumor during breast needle biopsy procedures, improving the outcomes. The model was shown to predict reasonably well the displacement by compression of lesions bigger or equal to 5 mm. Focusing in distinguish the different types of duct pathologies, Paul et al. (2022) used FE modeling to conduct a quantitative high-frequency US analysis. They were able to identify different duct pathologies through the peak density and the mean-peak-to-valley distance.

Besides compression, indentation has been also studied using FE analysis. In 1997, Zhang et al. (1997) developed a non-linear FE model to investigate the mechanics of indentation on soft tissues, especially the effect of friction and large deformation on the calculation of the Young’s modulus. The authors concluded that when the ratio between the radius of the indenter (a) and the thickness of the sample (h) is large (i.e., a/h = 2), the friction has a significant impact on the results. The same was concluded when considering high Poisson’s ratio (i.e., *ν* = 0.5). In the following year, Krouskop et al. (1998) also simulated an indentation test, using a tissue slice that was assumed as homogeneous since the tumor region was significantly larger than the indenter surface. Through the force-displacement curve, the Young’s modulus was calculated and the results showed that it did not depend on the loading frequencies, but its value increased with precompression.

Also in 1998, Erkamp et al. (1998) used a cylindrical soft tissue sample and place it in a mold, filling the spaces between the sample and the mold with a mixture of gelatine and agarose to prevent movement of the sample. Indentation tests were performed and the slope of the force-displacement curve was converted to the Young’s modulus using a conversion factor, which was obtained experimentally and validated by FE models. The maximum error obtained between the theoretical and experimental conversion factors was 14%, corresponding to the stiffer samples. Based on the approach developed by Erkamp et al. (1998), Samani et al. (2003), in 2003, proposed a reproducible measurement of the stiffness of small block samples of normal breast tissue. Indentation tests were performed and the slope of the force-displacement curve was converted into the Young’s modulus using a FE model. The FE simulation and the experimental results only diverged 3%. They witnessed a significant non-linear behavior, which indicates that Young’s modulus is sensitive to precompression. The same group, in 2007, Samani et al. (2007) created a FE mesh with the exact geometry of each sample of the normal breast tissue that was tested. The FE model simulated the indentation test (quasi-static loading, with an indentation of 0.5 mm) of each tissue specimen with an arbitrary Young’s modulus and a Poisson’s ratio equal to 0.495. In the end, the force-displacement slope was obtained. According to the equation *E* = *kS* (where *k* is the adimensional conversion factor, being the ratio of the arbitrarily input Young’s modulus and the calculated slope, in the simulation) the conversion factor was multiplied by the experimental measured force-displacement slope (*S*), obtaining the final tissue Young’s modulus (*E*). They observed that under small strains, fat and fibroglandular tissues had identical mechanical properties. When compared with pathologies, tumors had a higher Young’s modulus than fibroglandular tissues. In the same year, Samani and Plewes (2007) determined the properties of a breast tissue slice with a tumor. The FE method used in this work differs from the previous one regarding the conversion of the slope of the force-displacement curve into the Young’s modulus. In this situation, the method was performed iteratively using a tissue slice model. This method was validated by numerical simulations and experimental phantom studies. They concluded that the proposed FE method was robust and highly accurate to calculate the stiffness of the breast tissue samples.

Investigating also the hyperelastic behavior, Omidi et al. (2014) calculated the linear and hyperelastic parameters of DAT and normal breast tissues using the approach proposed in the studies of Samani et al. With the hyperelastic parameters, they simulated the DAT to evaluate its suitability as a breast implant. In addition, with MR images, they simulated a model of the human breast under gravity loading to predict the deformation associated with a change from the prone to supine position. They concluded that the best models for breast DAT were Yeoh and Ogden models, compared with first-order polynomial and Arruda–Boyce models, so the breast model was developed using breast-derived DAT with the Yeoh model. In another study, Haddad et al. (2016) used the FE model with DAT to evaluate the shape and deformation of the tissue under physiological loading conditions, not only from prone to supine position but also from prone to the upright position. The mesh was created slice by slice, using 8-noded hexahedral elements by the transfinite interpolation technique. They demonstrated that breast reconstruction using DAT had a similar deformation to normal breast under the same loading conditions.

Focusing on breast augmentation, Roose et al. (2005) developed a model to predict the postoperative shape of the breast (1 cm of accuracy) after a subglandular breast implantation. They compared a mass-spring system with a FE model, investigated the effect of different elasticity models, and evaluated different imaging modalities for the generation of patient-specific data. The validation showed that the errors were larger at sites where stresses were high, this could be explained by the biological effect of growth and atrophy, not considered in the FE models.

Another application of FE models is the study of bras, including the different designs, and investigating their mechanical impact on the breast. Sun et al. (2019) simulated the breast-shaping effect and the pressure distribution on the skin due to the bra. Moreover, bra-wearing was also simulated and good agreement with reality was achieved. In 2021, the same group studied different materials for the bra cup. Sun Y. et al. (2021) simulated the deformation of the breast and evaluated it in terms of the amount of uplifting and gathering of the breasts. With these results, the distribution of the contact pressure was obtained. They found that the stiffer material provided a better shape and low pressure in the straps, however, the pressure was high in the bottom part of the breasts. On the other hand, soft and flexible materials could reduce the pressure at the bottom of the breasts but they did not provide enough support which increased the pressure in the straps. They obtained an optimal elastic modulus for the bra cup material of 1.5 MPa since after this value no changes were observed in the uplifting and gathering of the breasts. Also focusing on the design of bras, Zhang et al. (2022) simulated elderly breast deformation under arm abduction, which was validated with motion data. The model included the torso, breast (defined as non-linear material), pectoralis major muscle, and rigid bones. The authors also created a questionnaire to understand the discomfort positions in a sports bra for elderly women as complementary information to design more ergonomic sports bras.

One of the drawbacks of FE models is the computational time, which is not realistic and viable for clinical practice. FE models are computationally intense regardless of the optimization methods, consuming a large amount of time due to the great number of nodes of the mesh as well as to the large geometry of interest compared to the area of interest (i.e., tumor) (Jeremic, 2021). It was reported in literature a computational time of 120 min to simulate the deformation of a breast under compression (Hopp et al., 2013; Solves-Llorens et al., 2014) Therefore, to overcome this issue, authors have been using machine learning along with FE models, using the simulations to train the algorithm before processing the patient’s data (Jeremic, 2021). Machine learning models. Rupérez et al. (2018) simulated the real-time breast compression (Neo-Hookean model for fat and fibroglandular tissues) and achieved computational times between 0.05 and 0.43 s. Martínez-Martínez et al. (2017) also simulated breast compression in real-time using the Mooney-Rivlin model for skin and adipose and glandular tissues and obtained computational times lower than 0.2 s. Both studies achieved computational times suitable for clinical practice.

### 4.2 Finite element models: 3D scaffolds

FE models can be a useful auxiliary tool for the 3D printing process as well. It is possible to study, in advance, different structures and evaluate the mechanical behavior of the scaffolds, which is an important step, first, to select the best geometry, porosity, and nozzle diameter without any fabrication (Sala et al., 2021) and, also, to promote tissue regeneration (Hendrikson et al., 2017).

Soufivand et al. (2020) and Schipani et al. (2020) used FE simulations to investigate the porosity of PCL scaffolds with different architectures, before 3D printing. The design will determine the porosity, which influences cell migration, the delivery of nutrients, and the removal of cell waste. Moreover, the design will also influence the mechanical behavior of the scaffold, which is important for cell differentiation. Soufivand et al. (2020) predicted the compressive mechanical properties and showed that the numerical results and the experimental results were in good agreement. The mechanical properties of the scaffolds were also modeled by Schipani et al. (2020), using FE modeling to capture the geometry and material behavior of printed scaffolds. This was also the goal of Sala et al. (2021), which printed PCL scaffolds with different geometries, porosity, and nozzle diameters and, then, using FE modeling determined the mechanical properties of each combination. The authors obtained structures that were capable of bearing different compressive and shear loads and with tunable porosity, with good quality and accuracy.

Castilho et al. (2018) also developed FE models as a tool to aid the design of 3D constructs, using a fiber scaffold (PCL) within a hydrogel matrix (GelMA). The material properties were obtained experimentally by compression. They developed a FE model (fiber scaffold: linear elastic; hydrogel matrix: Neo-Hookean model) based on idealized scaffold geometry and a micro-FE model (homogeneous linear elastic) based on micro-CT images. They studied the effects of reinforcement and load transfer, concluding that in scaffolds with higher volume fractions, the reinforcement mechanism was dominated by the load-carrying ability of the fiber interconnections.

Also using PCL but focusing on breast tissue regeneration, Liu et al. (2021) proposed an internal-bra-like prototype and used FE analysis to investigate the mechanical properties, specifically the stiffness. They obtained a stiffness similar to breast tissue and concluded that the introduction of more layers of mesh, results in a lower elastic modulus. At a microscopic level, the simulations indicated that cells experience heterogeneous mechanical stimuli at different places in the scaffold and that the local mechanical stimulus is controlled by the elastic modulus.

Ryan et al. (2009) developed three different titanium scaffolds, with different porosity. To characterize the mechanical performance of the scaffolds, macroscale, and unit-cell models were created based on micro-CT. They applied FE analysis and compared the results with experimental tests. It was demonstrated that the models could predict well the Young’s modulus and yield strength of the scaffolds. Using hydroxyapatite and calcium phosphate, Bagwan et al. (2021) also investigated the Young’s modulus and porosity of 3D printed scaffolds and concluded that decreasing the porosity, the strength increases and *vice versa*. The stiffness was also investigated using FE analysis by Yang et al. (2021), who concluded that by decreasing the wall thickness, the scaffold becomes brittle and tends to collapse, although by increasing the number of domains, the stiffness of the scaffold increases.

Focusing on the stiffness as well, Naghieh et al. (2018) investigated the effect of cross-linking on the mechanical properties of 3D-printed alginate scaffolds. Compression tests were carried out to measure the stiffness of the scaffolds and, then, a FE model was developed to predict the scaffold’s mechanical behavior. The authors concluded that both cross-linking time and volume of the cross-linker were important in the modulation of the mechanical properties of the scaffolds. Also using alginate, Kakarla et al. (2022) investigated the maximum stress regions and observed that the stress regions were at the soft zones near the pore area. Compared to experimental results, the stress-strain curves were similar, with a maximum strength obtained at 2.8 MPa for the experimental results and 2.7 MPa for the FE analysis. Zhou et al. (2016) focused on the gelation of a polymer, which is a complex process that involves chemical reactions and phase transitions, from a viscous fluid to a viscoelastic solid. They used agarose droplets, studying the temperature- and time-dependent degree of gelation and the deformation of the droplets during the process. They presented a model that could describe correctly the gelation process and predict the shear-stress distribution and deformations of the gel.

Investigating the non-linear behavior of polyvinyl alcohol hydrogel, Nazouri et al. (2020) used a FE algorithm and stress-relaxation data. The Mooney-Rivlin and Neo-Hookean strain energy functions, in which shear and bulk moduli vary with time, were applied and the results showed that the first function fitted better the stress-relaxation experiments. They concluded that polyvinyl alcohol hydrogel is a good cartilage substitute for tissue engineering therapies.

### 4.3 Finite element analysis: Final remarks

In literature, FE analysis has been used to simulate the breast and, in this way, predict its mechanical behavior and deformation. Studying the effect of mammographic compression, the impact of silicone implants, or the best design for bras are some investigations carried out in research. Moreover, FE modeling is also being used as a tool to predict the behavior of tissue engineering solutions. With FE approach, it is possible to study different geometries and architecture for 3D printed scaffolds. The influence of porosity and nozzle diameters are some examples of parameters that can be investigated prior to scaffold fabrication. Therefore, FE analysis allows the optimization of the 3D printing process. In addition, it is possible to simulate the implantation *in situ* of those solutions assessing the mechanical and physical impacts on the breast.

## 5 Discussion and conclusion

### 5.1 Breast tissue

Elastography is a common technique in *in-vivo* experiments to study breast tissues. Some authors only studied normal breast tissues, such as adipose and glandular tissues, while others also focused on diseased tissues, such as benign or malign tumors. There are different types of elastography, such as US, MR, or optical coherence tomographic elastography, however, the conclusions are similar independent of the technique used. Comparing only normal tissues, it is coherent that glandular tissue is stiffer than adipose tissue (Lawrence et al., 1998; McKnight et al., 2002; Van Houten et al., 2003; Chen et al., 2013). Moreover, when diseased tissues are taken into account, it is shown that the malignant tissue presents the highest stiffness, preceded by the benign tissue, and the normal tissue is the softest (Sinkus et al., 2000, 2005; Xydeas et al., 2005; Srivastava et al., 2011; Chen et al., 2013; Sayed et al., 2013). Another important conclusion is that the normal tissues of women with breast cancer are stiffer than the tissues of healthy women Chen et al. (2013). The observation of the viscoelastic behavior and hysteresis effect indicates that preconditioning is needed when investigating the mechanical properties of breast tissues Han et al. (2003). In terms of clinical applications, a precompression of around 10% is ideal to have better accuracy in distinguishing normal surrounding tissues from tumors Barr and Zhang (2012).

In terms of *ex-vivo* experiments, the mechanical tests can be performed using compression or indentation and might be used along with FE modeling. The general conclusions found in the literature are in accordance with the ones of *in-vivo* experiments, i.e., the malignant tissue (IDC) is the stiffer, and normal tissues are the softest (Sarvazyan et al., 1995; Krouskop et al., 1998; Wellman et al., 1999; Samani et al., 2003, 2007; Matsumura et al., 2009; Umemoto et al., 2014). Also, the stiffness of the tissues increases as the precompression increases, which proves the non-linear behavior of these tissues (Krouskop et al., 1998; Wellman et al., 1999; Samani et al., 2003; Matsumura et al., 2009; Umemoto et al., 2014). Comparing adipose breast tissue and adipose abdominal tissue, the elastic properties are similar but the viscous behavior is different (Calvo-Gallego et al., 2020). Moreover, the stiffness of normal breast tissues and DAT is similar (Omidi et al., 2014).

However, a wide range of values for the mechanical properties of breast tissues can be found in the literature. Considering the mechanical properties, normal breast tissues are softer than pathological tissues. Moreover, more invasive tumors are associated with increased stiffness (e.g., Young’s modulus). In literature, The variation in the elastic modulus is not only concerning the different types of breast tissues but also within each type of tissue. These differences might be caused by numerous factors, such as:

• Reduced number of samples and different locations where the tissue samples are removed
• Tissue heterogeneity and etiologic factors, such as age, hormonal state, menopause, pregnancy, etc.
• Systematic errors associated with the measurement techniques
• Different experimental protocols: testing parameters (e.g., speed, stress, and strain amplitude) and/or room conditions (e.g., temperature and humidity) and/or the mathematical approach
• Precompression and preconditioning, which has been different between studies: different levels of strain/stress and/or the number of cycles. Krouskop et al. (1998) reported higher values of Young’s modulus for the breast tissues and that can be explained by the larger preload compression (5% and 20%) applied in their tests.

However, the differences are not only found in *ex-vivo* experiments. Also in *in-vivo* experiments, the results might vary between studies. Even though authors used similar imaging techniques, for example, the different shear wave frequencies applied in the MR elastography, might lead to different results.

A limitation of the *ex-vivo* experiments, compared to *in-vivo*, is that none of the studies consider the effect of gravity, hydration, and tissue fibers as it happens in *in-vivo*, where blood supply and interstitial fluids exist. Even though researchers try to make the sample hydrated, the absence of the other fluids might contribute as well to the differences found in literature (Ramião et al., 2016).

Comparing the two experimental tests, *ex-vivo* is the most suitable to study large deformations, since *in-vivo* data is only collected under small precompression. In elastography, the results are highly dependent on the precompression applied, influencing the outcomes, such as the distinction between a benign and malignant tumor (Matsumura et al., 2009). showed clearly this fact, by reporting that DCIS is difficult to detect when high compressions are applied during elastography, which can easily induce false negatives.

Therefore, there is a need to quantify the ideal precompression for the different types of breast tissue. Moreover, better knowledge concerning the hyperelastic behavior of the tissues must be achieved as well, in order to improve the clinical diagnostic techniques as well as surgical approaches.

### 5.2 Breast tissue scaffolds

Improving the knowledge about the mechanical properties of breast tissues, will enhance the outcomes in the breast tissue engineering field. Researchers have been combining biomaterials with autologous cells to propose solutions for breast tissue regeneration as an alternative to the current clinical approaches such as silicon implants and autologous tissue. Summing up, some advantages of tissue engineering approaches are (Janzekovic et al., 2020):

• Use of additive manufacturing in the scaffolds’ design and fabrication
• Many design solutions, according to the desired degradability, resorbability, and biocompatibility
• Different shapes, volumes, and structures. A porous morphology is achievable, addressing the limitations of mass transfer and mechanical properties
• Easily reproducible and patient-specific solutions
• Controllable chemical, mechanical and physical properties. For example, the mechanical properties can be tailored by modifying the ratio surface area/mass and the porosity, including pore size
• Flexible configuration to vary the surface area for cell attachment and to optimize nutrients availability and waste transports
• Possibility to incorporate antibiotics and chemotherapy drugs

One of the most common surgical approaches is breast reconstruction and augmentation. The current clinical approach includes the use of autologous tissue and silicone implants, which have their own limitations. Tissue engineering is being investigated as a better alternative, corresponding to the combination of synthetic or natural materials with autologous cells (Cleversey et al., 2019).

The ideal scaffold would have the modifiable mechanical properties of synthetic biomaterials and the biomimetic properties of naturally occurring biomaterials (O’Halloran N. A. et al., 2018). However,Janzekovic et al. (2020) defends that the ideal scaffold is unlikely to exist since a tissue might have multiple function roles, which could not be accomplished with a single universal scaffold. The scaffold should provide mechanical support and biochemical cues to promote cell adhesion, proliferation, migration, and differentiation (O’Halloran N. A. et al., 2018). Typically, the scaffold provides the necessary architecture and vasculature for long-term stability and viability. Moreover, it should be biodegradable, allowing its replacement overtime by the new tissue.

For adipose tissue regeneration, scaffolds are often seeded with ADSCs, which present advantages compared to other cells, such as the high capability of differentiation, including the production of factors that promote vascularization, tissue growth, immune modulation, and cell recruitment (Donnely et al., 2020). However, one of the problems of cell therapies is the unknown degree of possible cancer recurrence (Combellack et al., 2016; Donnely et al., 2020), since the cell mechanisms to generate normal or diseased tissue are the same. Therefore, it is crucial that the cells’ differentiation occurs into the desired lineage, which can be manipulated through the scaffolds and their properties. Another challenge of breast tissue regeneration concerns the large volume of adipose tissue needed (O’Halloran N. A. et al., 2018), which depends on vascularization for tissue survival (Cleversey et al., 2019). Moreover, the implantation of scaffolds introduces some risk factors, such as graft survival, volume loss, and fat necrosis (Donnely et al., 2020). There is also the possibility of scar formation and the increase of radio-density (Janzekovic et al., 2020), which can be overcome with the use of degradable porous scaffolds, being less radiopaque, causing less scar tissue and, after degradation, not interfering with imaging techniques (Visscher et al., 2017). An alternative to the implantable scaffolds is the injectable scaffolds, which have the advantage of being minimally invasive for the patient, being a low morbidity procedure, and filling the defect site more accurately (O’Halloran N. A. et al., 2018; Donnely et al., 2020).

The most common biomaterials in adipose tissue regeneration are hydrogels since they accurately mimic the adipose ECM and they effectively encapsulate the cells (O’Halloran N. A. et al., 2018). Materials such as alginate (Cavo et al., 2016), gelatin and its modifications (Ovsianikov et al., 2010; Van Hoorick et al., 2015; Salamon et al., 2014; Zigon-Branc et al., 2019; Markovic et al., 2015; Tytgat et al., 2019a, b; Sutrisno et al., 2021), polyurethane (Zhou et al., 2020), PEGDA (Aliabouzar et al., 2018), PCL (Chhaya et al., 2016; Rocco et al., 2019; Griffin et al., 2020; Meng et al., 2020, 2021; Cheng et al., 2021) and collagen (Puls et al., 2021) are some of the material reported in the literature and that are being studied for breast tissue regeneration since their properties are similar to the breast tissues. These materials have been reported to support cell adhesion, proliferation, and differentiation of MSCs or ADSCs. It has been stated that softer substrates have better outcomes than stiffer materials (Cavo et al., 2016; Aliabouzar et al., 2018; Zigon-Branc et al., 2019) and that the ideal pore size is between 500 and 1,000 *μ*m (Tytgat et al., 2019b). Some authors used fat injection allied to the scaffold, reporting better results than when the scaffolds were used alone (Chhaya et al., 2016; Maxwell and Gabriel, 2016; Rocco et al., 2019). Also, others produce scaffolds with DAT (Cheung et al., 2014) or ECM components (Sokol et al., 2016), reporting good results in terms of viability, adipogenesis, and tissue formation. With these materials, 3D printing is a promising technology for scaffold production, that allows the creation of precise and reproducible 3D constructs that mimic the native tissues. Moreover, large volumes can be produced, at a scale viable for clinical applications (Cleversey et al., 2019).

However, more research needs to be done in order to create the optimal patient-specific scaffold that accurately mimics the 3D architecture and mechanical properties of the native breast adipose tissue, promoting angiogenesis and adipogenesis for large-volume reconstructions, and ensuring the safety of the patient.

### 5.3 Finite element analysis

A complementary approach to investigate the mechanical properties of breast tissues, as well as the 3D printed scaffolds, is FE modeling. With FE models, it is possible to study the mechanical properties of breast tissues, including the hyperelastic properties and Cooper’s ligaments. Also, it allows studying the mechanical properties of the tissues under different situations and conditions, matching women’s daily routines, such as walking, jogging, laying down, etc. With FE models, the time of the study compared to the experimental approach in which a relevant number of volunteers would be needed. Moreover, by coupling FE with machine learning, the computational time can be decreased.

FE modeling is being used as a tool to analyze and evaluate the mechanical behavior of the breast. Some authors use this technique not only to model the breast but also to include silicone implants and evaluate their impact. Commonly, the authors resort to US, CT, or MR images to model the breast and defined it as an isotropic and homogeneous material (Danch-Wierzchowska et al., 2018; Chen et al., 2019). Studies defined the breast as a linear elastic material (Danch-Wierzchowska et al., 2018), while others defined as hyperelastic material (Samani a. et al., 2001), using Mooney-Rivlin (Liu et al., 2017; Martínez-Martínez et al., 2017; Myung et al., 2019), Neo-Hookean (Rupérez et al., 2018; Chen et al., 2019; Na et al., 2019) and Ogden models (Sun et al., 2021c, b).

In case there is an implant, the authors have been studying the deformation of the breast under gravity (Dong et al., 2016) and from prone to supine position (Danch-Wierzchowska et al., 2018), and the stress variations (Myung et al., 2019). As an alternative to the silicone implants, DAT is also been simulated as linear and hyperelastic material and the deformation was assessed from prone to supine positions (Omidi et al., 2014) and from prone to upright positions (Haddad et al., 2016). Nevertheless, also without an implant, changes on the breast from prone to supine positions are been investigated (Chen et al., 2013) as well as the breast under compression (i.e., mammography) (Sturgeon et al., 2016; Liu et al., 2017) or indentation (Zhang et al., 1997). FE modeling has also been used coupled with *ex-vivo* compression and indentation experimental studies, as a tool for validation or analysis of the experimental results (Erkamp et al., 1998; Krouskop et al., 1998; Samani et al., 2003; Samani et al., 2007; Samani and Plewes, 2007). It also can be used to simulate the postoperative shape of the breast after augmentation (Roose et al., 2005) as well as the impact of the bras in the breast (Sun et al., 2019).

FE modeling can be a helpful tool to aid physicians in clinical practice, for example, to predict the location of a tumor (Azar et al., 2001). However, the computational times are not realistic. Putting together FE modeling and machine learning, it is possible to have real-time breast compression and its outcomes in a period of time suitable for clinical practice (Martínez-Martínez et al., 2017; Rupérez et al., 2018).

Besides breast tissues, also scaffolds have been investigated using FE modeling, for example, to study their porosity (Schipani et al., 2020; Soufivand et al., 2020), mechanical performance (Ryan et al., 2009; Meng et al., 2020, 2021; Nazouri et al., 2020), gelation process (Zhou et al., 2016) or crosslink effect (Naghieh et al., 2018). On the other hand, FE modeling can be helpful as a tool to aid the 3D printing process (Castilho et al., 2018). When studying the different biomaterials, authors might define them as linear elastic materials or hyperelastic materials. If the authors consider hyperelastic properties, Mooney-Rivlin and Neo-Hookean are the constitutive models commonly used (Castilho et al., 2018; Nazouri et al., 2020). The FE modeling is not only helpful to optimize the printing process by prior testing the different combinations but also to predict the behavior of the printed scaffold under different conditions such as at the implantation site.

For the case of 3D printing and scaffold production, FE models may help in the optimization of the 3D printing process, decreasing the experimental time when the trial-error approach is used. Also, with FE models it is possible to study the mechanical properties of the scaffolds virtually, testing different mixtures, for example,. Moreover, FE models are a very valuable tool to predict the behavior of the scaffold under different conditions, including at implantation time.

Therefore, FE modeling is a powerful tool that should be used in parallel to experimental studies, in order to optimize the outcomes.

## Abbreviations

3D, Three Dimensional; ADM, Acellular Dermal Matrices; ADSCs, Adipose Derived Stem Cells; CT, Computed Tomography; DAT, Decellularized Adipose Tissue; DCIS, Ductal Carcinoma *in situ*; ECM, Extracellular Matrix; FDM, Fused Deposition Modeling; FE, Finite Element; GelMA, Gelatin Methacrylated; IDC, Invasive Ductal Carcinoma; MR, Magnetic Resonance; MSCs, Mesenchymal Stem Cells; PCL, Polycaprolacton; PEGDA, Polyethylene Glycol Diacrylate; PLA, Polylactic acid; US, Ultrasound.
